# Engineering the Modular Receptor-Binding Proteins of *Klebsiella* Phages Switches Their Capsule Serotype Specificity

**DOI:** 10.1128/mBio.00455-21

**Published:** 2021-05-04

**Authors:** Agnieszka Latka, Sebastien Lemire, Dennis Grimon, Dorien Dams, Barbara Maciejewska, Timothy Lu, Zuzanna Drulis-Kawa, Yves Briers

**Affiliations:** a Department of Biotechnology, Ghent University, Ghent, Belgium; b Department of Pathogen Biology and Immunology, University of Wroclaw, Wroclaw, Poland; c Synthetic Biology Group, Massachusetts Institute of Technology, Cambridge, Massachusetts, USA; d Department of Electrical Engineering and Computer Science, Massachusetts Institute of Technology, Cambridge, Massachusetts, USA; e Department of Biological Engineering, Massachusetts Institute of Technology, Cambridge, Massachusetts, USA; f Research Laboratory of Electronics, Massachusetts Institute of Technology, Cambridge, Massachusetts, USA; g Broad Institute, Cambridge, Massachusetts, USA; h Antimicrobial Resistance Interdisciplinary Research Group, Singapore-MIT Alliance for Research and Technology, Singapore; Universidade de Sao Paulo

**Keywords:** bacteriophage, *Klebsiella*, depolymerase, horizontal transfer, receptor-binding protein

## Abstract

The high specificity of bacteriophages is driven by their receptor-binding proteins (RBPs). Many *Klebsiella* bacteriophages target the capsular exopolysaccharide as the receptor and encode RBPs with depolymerase activity. The modular structure of these RBPs with an N-terminal structural module to attach the RBP to the phage tail, and a C-terminal specificity module for exopolysaccharide degradation, supports horizontal transfer as a major evolutionary driver for *Klebsiella* phage RBPs. We mimicked this natural evolutionary process by the construction of modular RBP chimeras, exchanging N-terminal structural modules and C-terminal specificity modules. All chimeras strictly follow the capsular serotype specificity of the C-terminal module. Transplanting chimeras with a K11 N-terminal structural RBP module in a *Klebsiella* phage K11 scaffold results in a capsular serotype switch and corresponding host range modification of the synthetic phages, demonstrating that horizontal transfer of C-terminal specificity modules offers *Klebsiella* phages an evolutionary highway for rapid adaptation to new capsular serotypes.

## INTRODUCTION

Bacteriophages infect bacteria with high specificity. A major determinant for this specificity is located in the phage-encoded receptor-binding proteins (RBPs) that structure phage tail fibers and/or tail spikes. RBPs mediate the initial contact of the phage with their cognate receptors on the host cell surface and are essential to initiate the infection process (1–3). Many *Klebsiella* phages target the capsular exopolysaccharide as an initial receptor. Their RBPs show depolymerase activity, actively binding and degrading the capsule (4). *Klebsiella* strains show a large capsular diversity with at least 79 serotypes reported today, each having a different polysaccharide composition (5), and the RBP diversity is correspondingly high. Phage RBPs are generally specific for a single (or a few) serotype(s), and the relationship between RBP specificity, capsular serotype, and phage host spectrum has been extensively demonstrated for wild-type phages (6–15). The number of RBPs with depolymerase activity per phage is highly variable. Phage KP36 produces a single RBP and is only active against serotype K63, corresponding to the substrate specificity of its RBP (8, 16), whereas other phages (e.g., KP32, K5-2, K5-4, and ΦK64-1) encode more RBPs with depolymerase activity, recognizing a corresponding number of capsular serotypes (6, 7, 9, 10, 17) and resulting in a broader host spectrum. A similar relationship has also been described for Escherichia coli and *Salmonella* phages (18–20).

*Klebsiella* phage RBPs typically have a modular structure with N-terminal module(s) dedicated to structural organization and attachment of the RBP to the phage tail structure, while C-terminal modules are generally involved in receptor binding and degradation (16). Some RBPs comprise an additional C-terminal chaperone for proper folding and trimerization, followed by autoproteolytic cleavage (21–25). This modular build-up of RBPs is shaped by intense horizontal transfers (26, 27). Further accumulation of mutations (vertical transfer) has magnified the diversity of the polysaccharide-degrading enzymatic domains (4, 19, 23, 28–30). In our previous work, the RBP architectures of 59 *Klebsiella* phages spread over six taxonomic groups have been analyzed along with suggested evolutionary linkages between the different RBP organizations (16). Horizontal transfer appears to be the major evolutionary force to drive host adaptation in a broad range of *Klebsiella* phages. To mimic this natural evolutionary process on a laboratory scale, we constructed and evaluated modular chimeras, exchanging N-terminal structural modules and C-terminal specificity determinants. In addition, we constructed synthetic phages with these functional chimeras, confirming that horizontal transfer can drive serotype specificity and host range switching in *Klebsiella* phages.

## RESULTS

### The capsule serotype specificity of chimeric RBPs follows the C-terminal depolymerase domain.

*Klebsiella* phages KP32, KP34, and KP36 were selected as representatives of well-characterized *Klebsiella* phage groups. Their RBPs with depolymerase activity were identified and characterized previously (8, 9, 31), and their RBP architectures differ significantly (16) (see Fig. 2A). Phage KP32 has two RBPs, with a first RBP connected to the phage tail via an N-terminal anchor domain (1A), while the second RBP (2E) interacts with the first RBP (31). Podovirus KP34 has a single RBP (3E) indirectly attached to the virion via an intermediate anchor protein (3A), and siphovirus KP36 has a single RBP, including an N-terminal anchor domain (4A) for direct attachment to the phage particle. The two depolymerase domains of phage KP32 (1E and 2E) have a K3 and K21 capsular specificity, respectively, whereas the single RBP of phages KP34 (3E) and KP36 (4E) are both specific to the K63 serotype. The depolymerase domain of phage KP34 (3E) and phage KP36 (4E) share 42% identity in amino acid sequence. This high similarity suggests an evolutionary linkage across morphotype borders (podovirus/siphovirus), resulting from a horizontal transfer followed by vertical evolution through the accumulation of mutations.

**FIG 1:**
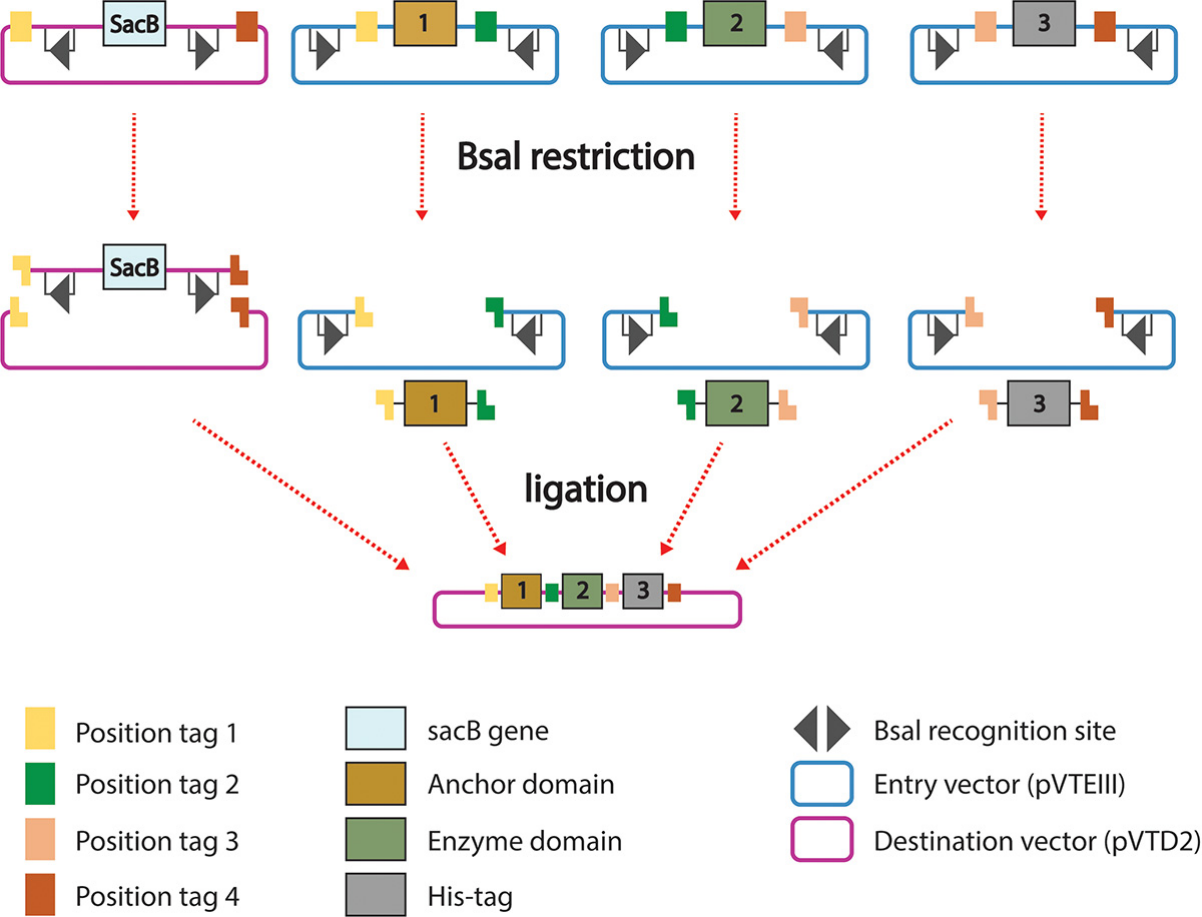
VersaTile method. Tiles provided with position tags corresponding to positions 1, 2, and 3 are cloned in the pVTEIII entry vector. Subsequently, they are mixed with the pVTD2 destination vector (possessing the *sacB* gene as a negative selective marker). This mixture is subjected to restriction (BsaI) and ligation (T4 DNA ligase) cycles in one tube. Tiles are subsequently assembled into the destination vector pVTD2 in the designed order according to the position tags.

**FIG 2:**
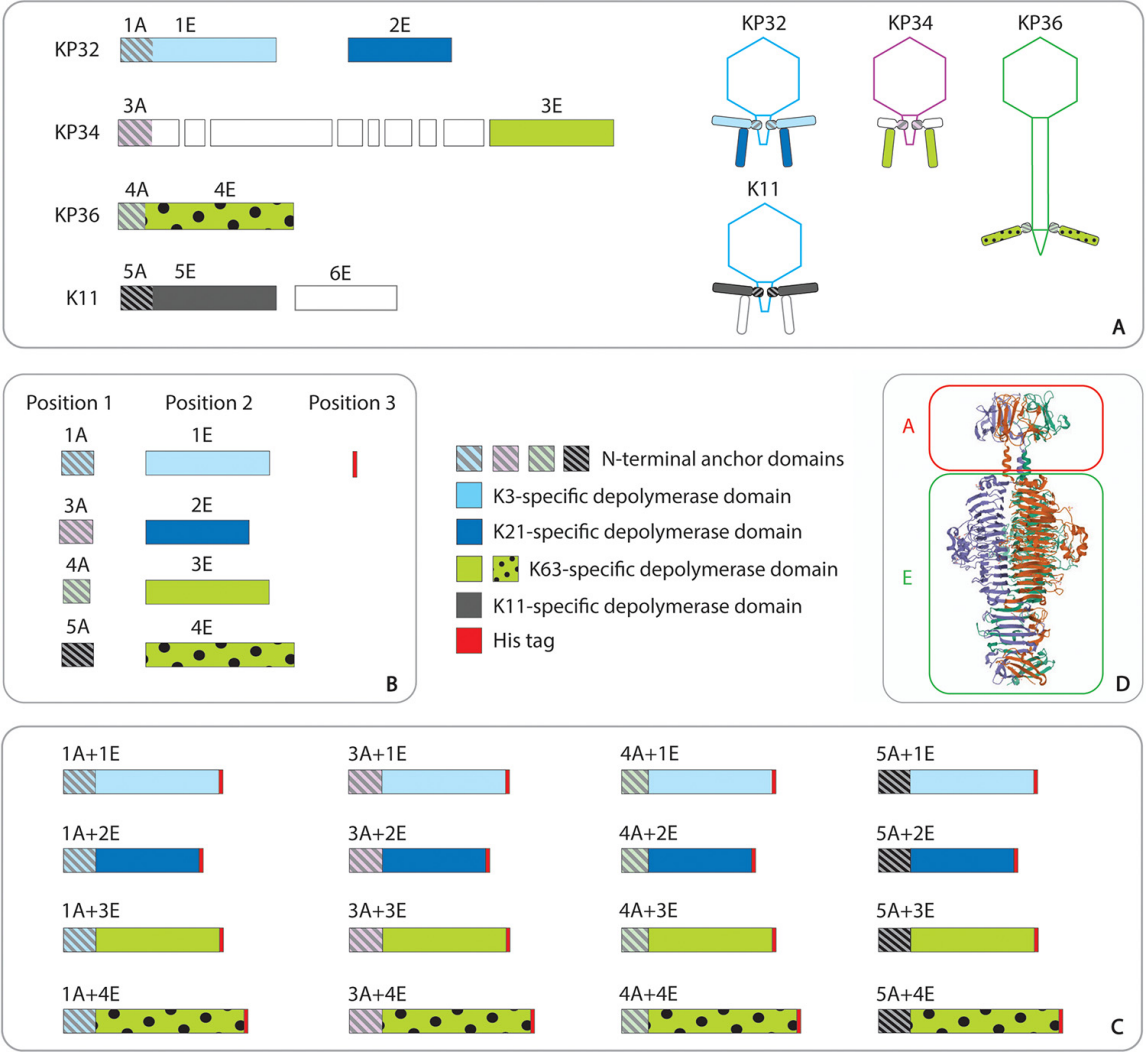
Modular architecture of *Klebsiella* phage RBPs and their chimeras. (A) Overview of the genomic context of the RBPs of phages KP32, KP34, KP36, and K11, along with their modeled RBP architectures. The different anchor domains are labeled A, preceded by the protein number. This A domain is predicted to anchor the RBP to the virion, and all anchor domains differ substantively in amino acid sequence, except for 1A and 5A, which share 88% similarity at the amino acid level. The enzymatic domains with depolymerase activity are labeled E, preceded by the protein number. The colors correspond to the capsule serotype specificity. The first and second RBP of phage KP32 are KP32gp37 (YP_003347555.1) and KP32gp38 (YP_003347556.1). The intermediate anchor protein and the RBP of phage KP34 are KP34gp49 (YP_003347643.1) and KP34gp57 (YP_003347651.1), respectively, whereas the single RBP of phage KP36 is KP36gp50 (YP_009226011.1). The white blocks in phage KP34 correspond to intervening, non-RBP genes. The RBP structure of phage K11 (belonging to group A of the KP32 viruses, *Przondovirus*; 16, 35–37) follows a pattern similar to that of phage KP32 with a first (K11gp17; YP_002003830.1) and second (K11 ASC_0043; YP_002003831.1) RBP. (B) Preparation of a tile repository. Each tile contains a coding sequence for either an anchor or enzymatic domain, flanked by position tags for position 1 (anchors), position 2 (enzymatic domains), and position 3 (hexahistidine tag for protein purification). (C) Overview of the chimeric RBPs combining cognate and noncognate anchor (position 1) and enzyme (position 2) domains, followed by a C-terminal hexahistidine tag (position 3). (D) The tail spike of *Salmonella* phage P22 (PDB entry 2XC1) illustrates a typical modular RBP structure and guided the construction of chimeras. The RBP has an N-terminal dome-like anchor domain (A), a central β-helical domain for host recognition and enzymatic activity, and a C-terminal domain responsible for protein trimerization and/or receptor recognition (E) (24, 54, 55). The tiles corresponding to the anchor domains (1A, 3A, 4A, and 5A) included both the N-terminal dome-like domain and the long α-helix that physically separates both functions of the RBP (red frame), while the tiles of the enzymatic domains 1E, 4E, and 5E combine the remaining parts (green frame). Phyre2 analyses were used to predict the long α-helix in 1A, 3A, 4A, and 5A. Based on this prediction, the anchor domain ends were delineated after 178, 170, 135, and 178 aa for 1A, 3A, 4A, and 5A, respectively.

Each possible chimera recombining an anchor domain (1A, 3A, and 4A) and an enzymatic domain (1E, 2E, 3E, and 4E) of the three different phages were produced, resulting in 12 combinations. In addition, we also produced four chimeras using the corresponding anchor domain (5A) of the well-studied K. pneumoniae phage K11. Phage K11 clusters taxonomically with phage KP32. The VersaTile technique (32) was used to produce the sequences for the chimeras, since this technique is dedicated to the convenient assembly of nonhomologous building blocks, such as anchor and enzymatic domains. A three-position design was chosen. At first, all building blocks to be recombined were converted to so-called tiles by flanking them with 6-nucleotide (nt) position tags. Anchor (1A, 3A, 4A, and 5A) and enzymatic (1E, 2E, 3E, and 4E) domain sequences are labeled for positions 1 and 2, respectively, whereas a hexahistidine tag was labeled for position 3 (Fig. 1 and 2B). The delineation of the anchor and enzymatic domains was guided by the crystal structure of the tail spike of *Salmonella* phage P22, showing a prominent physical separation between the anchor and enzymatic domain by an extended α-helix. Therefore, each anchor domain was delineated after its long α-helix, as in the P22 tail spike (Fig. 2D; see also Fig. S1 in the supplemental material). Secondly, all coding sequences of 16 chimeras were constructed by combining the respective tiles in a VersaTile assembly reaction (Fig. 2C). The used destination vector (pVTD2) was a high-copy-number vector with a T7 promoter for expression in E. coli.

10.1128/mBio.00455-21.1 FIG S1 Identification of long α-helices that separate anchor domains. The consensus secondary structures of the N termini of KP32gp37 (A), KP34gp49 (B), KP36gp50 (C), and K11gp17 (D) were predicted using Phyre2 (L. A. Kelley et al., 2015, Nat Protoc, https://doi.org/10.1038/nprot.2015.053). Long α-helixes that are hypothesized to physically separate the N-terminal dome-like domain and the enzymatic domain are indicated in red frames FIG S1, PDF file, 0.3 MB. Copyright © 2021 Latka et al. 2021 Latka et al. https://creativecommons.org/licenses/by/4.0/ This content is distributed under the terms of the Creative Commons Attribution 4.0 International license.

**FIG 3:**
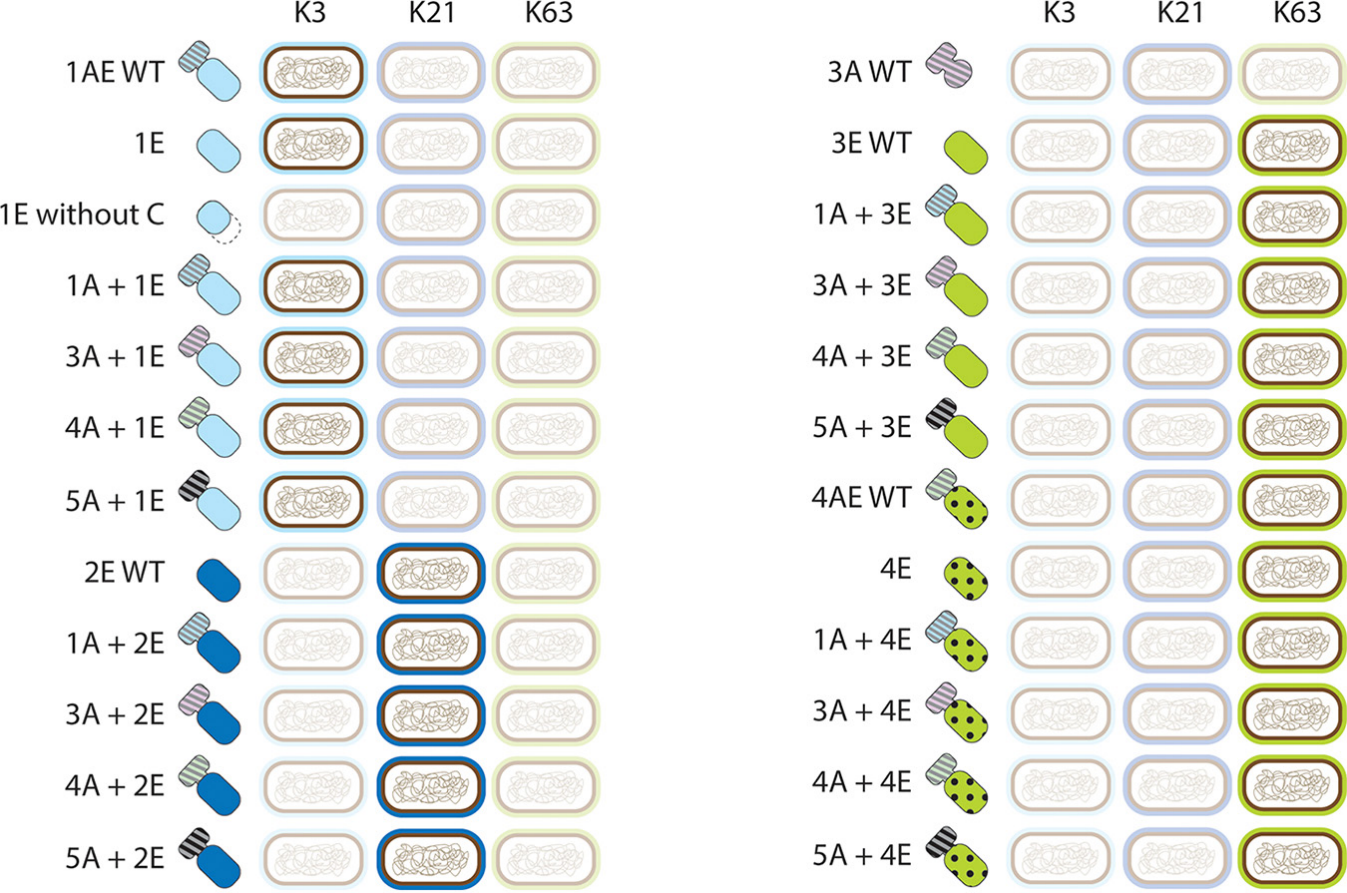
Capsule serotype specificity of chimeric RBPs. All chimeras and control proteins were expressed and examined on bacterial lawns of *Klebsiella* strains with the K3 serotype (host of phage KP32), K21 serotype (host of phage KP32), and K63 serotype (host of phages KP34 and KP36). The chimeric proteins (Table S3) and their modular structure are represented in different colors and shades. The presence of visible halos on a particular strain is highlighted. In all cases, there was a strict correlation between the enzymatic domain (when present) and the capsule serotype, except when the chaperone domain was deleted in 1E and when using the 3A wild-type protein, which has only an anchoring function and no enzymatic domain. A plus sign between domains in the code name means that the chimeric protein is the result of VersaTile assembly. When there is no plus sign between the anchor (A) and enzyme (E) domain (for example, 1AE WT), the wild-type (WT) depolymerase was prepared, as done for the native sequence, without a cloning scar between the anchor and enzyme domain. VersaTile-assembled chimeras have an intervening linker of two amino acids between both domains, resulting from the position tag.

All 16 chimeras were successfully expressed in a small-scale culture (10 ml) (Fig. S2 and Table S3). Lysates were spotted on lawns of K. pneumoniae clinical strains with defined capsular serotypes (K3, K21, and K63) to examine the enzymatic activity and specificity of the chimeric proteins (Fig. S3). Control proteins included purified stocks of the full-length wild-type RBPs of phage KP32 (1AE WT and 2E WT), phage KP34 (3A WT and 3E WT), and phage KP36 (4AE WT). In addition, the separate enzymatic domains (1E and 4E) were expressed. All spotted proteins caused a semitransparent halo, demonstrating decapsulating enzymatic activity, except for 3A WT, which lacks an enzymatic domain (Fig. 3). The capsular serotype specificity always followed the original specificity of the enzymatic domain of the chimera, regardless of the anchor fusion partner. For example, the 1AE wild-type protein and 1E domain are active on a strain with the K3 serotype, corresponding to the phage host specificity; consistent with this, all chimeras bearing 1E were only active on the K3 serotype as well. Enzymatic domains that have no natural anchor domain (2E and 3E) also retain their activity and capsule serotype specificity upon fusion to a noncognate anchor domain. Note that taxonomic borders were crossed in some chimera. For example, 1A + 4E combines an anchor domain of podovirus KP32 and an enzymatic domain of siphovirus KP36. This suggests that the evolution of RBPs can be successful by horizontal transfer across phage families.

10.1128/mBio.00455-21.2 FIG S2 Overexpression of chimeric depolymerases (CD), truncated receptor binding protein (T RBP), enzyme domain of depolymerases (E), and wild-type depolymerases (D) compared to that of BL21(DE3) cells transformed with plasmid without an insert as a control (C). Wild-type proteins were purified and purified protein is shown, while for all other proteins the lysate is shown as indicated in Table S3. M indicates the molecular mass marker (Precision Plus protein unstained standards, Bio-Rad, or Roti Mark standard, Carl Roth) is indicated. Definitions of the code and the respective molecular weights can be found in the Table S3. FIG S2, PDF file, 0.4 MB Copyright © 2021 Latka et al. 2021 Latka et al. https://creativecommons.org/licenses/by/4.0/ This content is distributed under the terms of the Creative Commons Attribution 4.0 International license.

10.1128/mBio.00455-21.3 FIG S3 Typing of a chimeric depolymerase on K. pneumoniae strains with different capsular serotypes, illustrated here for the chimeric depolymerase 1A + 3E (KP32gp37 anchor fused with the KP34gp57 depolymerase). Volumes of 10 μl lysate containing either the chimeric depolymerase or one of the controls were spotted on bacterial lawns of five different strains with three different serotypes (CD, chimeric depolymerase 1A + 3E; C, bacterial lysate with empty vector; B, lysis buffer used for lysate formation). The chimeric depolymerase remains specific to capsular serotype K63, similar to the specificity of the enzymatic domain from KP34gp57 (3E). Download FIG S3, PDF file, 0.2 MB. Copyright © 2021 Latka et al. 2021 Latka et al. https://creativecommons.org/licenses/by/4.0/ This content is distributed under the terms of the Creative Commons Attribution 4.0 International license.

10.1128/mBio.00455-21.7 TABLE S3 Composition of all chimeras with expected molecular weights (MW, kDa). In the case of KP32gp37 (1AE), which has a C-terminal autocleaving chaperone domain, the expected MW of the cleaved protein is given as well as the MW of the autocleaved moiety (12.3 kDa). Soluble protein fractions (SPF) were examined to check overexpression of chimeric depolymerases/wild-type (WT) depolymerase/enzymatic domains, except when protein overexpression was not visible in the SPF. In these cases, the total protein fraction (TPF), which also comprises insoluble proteins, was examined instead (Fig. S2). Fractions (SPF/TPF) marked with V are visualized with SDS-PAGE (Fig. S2). X indicates the presence of a particular domain in the chimeric depolymerase/wild-type (WT) depolymerase/enzymatic domain. A plus sign between domains in the code name means that the chimeric protein is the result of VersaTile assembly. When there is no plus sign between the anchor (A) and enzyme (E) domain, the wild-type (WT) depolymerase was prepared as in the native sequence without scar between the anchor and enzyme domain, whereas VersaTile assembled chimeras have an intervening linker of two amino acids between both domains, resulting from the position tag. Download Table S3, PDF file, 0.09 MB. Copyright © 2021 Latka et al. 2021 Latka et al. https://creativecommons.org/licenses/by/4.0/ This content is distributed under the terms of the Creative Commons Attribution 4.0 International license.

Since we observed that 1AE releases its C-terminal chaperone domain during expression by autoproteolytic cleavage, we also evaluated the 1E domain without its chaperone. The protein could be expressed but only in the insoluble fraction, and it did not show any enzymatic activity, stressing the role of the chaperone domain for correct folding (Fig. 3).

### Synthetic phages with chimeric RBPs are infective and follow the serotype specificity of the swapped RBP.

All chimeras were found to be enzymatically active, demonstrating that the delineation of domains did not disrupt the enzymatic activity. Moreover, the chimeric proteins were able to fold properly despite possessing a noncognate N-terminal anchor. Consistent with the prediction, the enzymatic specificity of the chimera strictly correlates to the specificity of its enzymatic domain. Thus, the enzymatic domains behave as true modules having an autonomous folding and autonomous function, two prerequisites for a successful horizontal transfer. To evaluate whether the anchor domains also conserve their autonomous folding and function, i.e., anchoring of the RBP to the phage particle, we constructed and evaluated synthetic phages with the original RBP replaced by a chimeric RBP. In addition, we assessed the presence (or absence) of capsular serotype specificity switches.

We used the phage engineering platform in S. cerevisiae based on the yeast gap repair system to assemble synthetic phage genomes in a yeast artificial chromosome (YAC), followed by electroporation of E. coli cells with the isolated YAC for rebooting of the assembled phage genomes (33). As a first step to evaluate this phage engineering platform for our purposes, we used isolated wild-type genomic DNA of *Klebsiella* phage KP32 for electroporation of E. coli 10G cells. The cells were lysed to release the rebooted synthetic phages, and the lysate was plated on the respective *Klebsiella* host strain. Plaques were formed thus, infective KP32 phages could be rebooted in a nonnatural host by electroporation. However, *in vivo* assembly of the same phage genome in YAC and subsequent rebooting was unsuccessful. One reason might be the low yield of successfully assembled YAC DNA in combination with a low transformation efficiency. Therefore, we used K. pneumoniae phage K11 as an engineering scaffold, the genome of which was successfully assembled and rebooted before (33) and in this study (Fig. S4). Phage K11 belongs to group A of *Przondovirus* and has a high identity at the genome level with phage KP32 (91% identity with a query coverage of 88%). One RBP (K11gp17; 5AE) with depolymerase activity against capsular serotype K11 has been reported before (34), but based on our previous *in silico* analysis, a second RBP (K11 ASC_0043; 6E) with a putative depolymerase activity was predicted (Fig. 2A) (16). Protein K11 ASC_0043 has been recombinantly produced, but its capsular specificity has not been found, despite screening a broad collection of *Klebsiella* capsular serotypes (*n* = 79). The first RBPs of phage K11 and KP32, K11gp17 and KP32gp37, respectively, share 88% identity in their N termini (157/178 amino acids [aa]). However, the remainder of the protein comprising the enzymatic domain is distinct, explaining why phage K11 does not infect KP32 host K. pneumoniae 271 (K3) but targets capsule serotype K11 instead.

**FIG 4:**
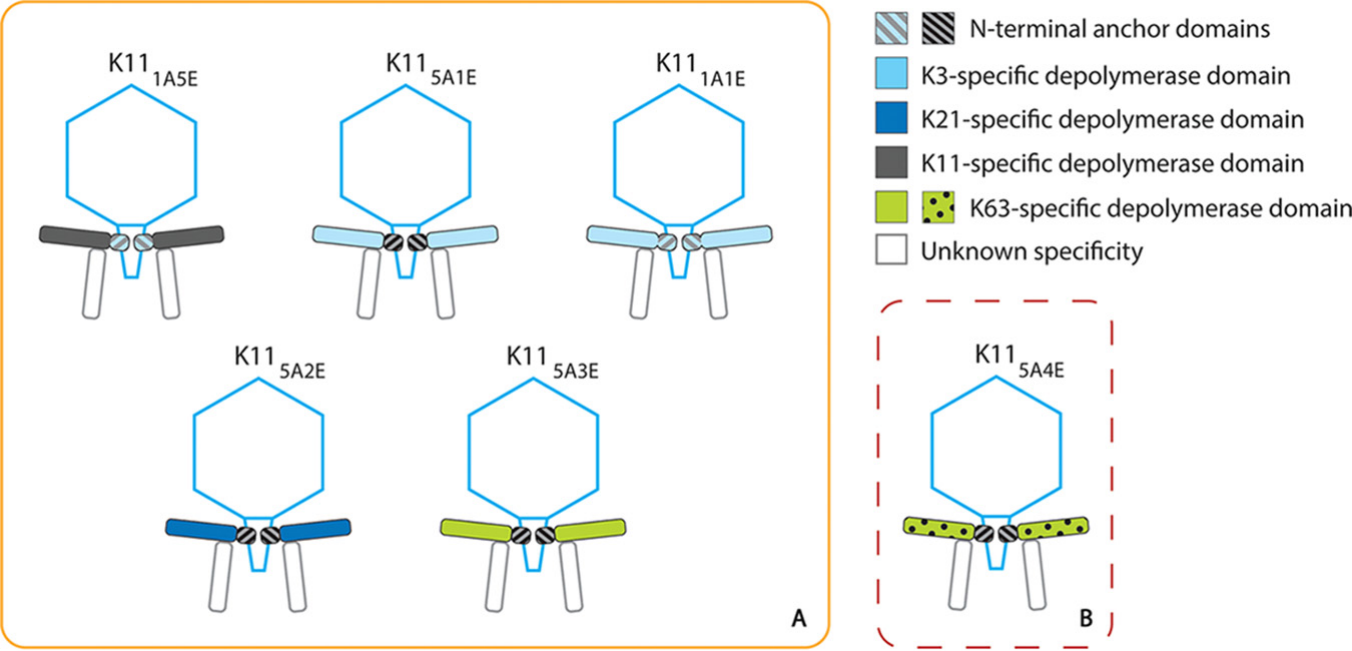
Modifications of phage K11. (A) Overview of synthetic K11 phages with a chimeric RBP constructed in this work in the order of appearance in the text. (B) Synthetic phage K11_5A4E_ could not be successfully rebooted. The subscripts indicate the chimeric RBP that replaces the native first RBP of phage K11 (K11gp17) (Tables S3 and S4). The second putative RBP of phage K11 is shown transparently because its presence could not be confirmed experimentally, as its receptor is unknown.

10.1128/mBio.00455-21.4 FIG S4 Representative plaques of rebooted phages using the phage engineering platform. Assembled wild-type K11 is plated on K. pneumoniae 390 (host of K11) (A), K11_5A3E_ (K11 with K11gp17 anchor fused with KP34gp57 depolymerase; Fig. 4) is plated on K. pneumoniae 77 (host of KP34) (B), and K11_5A1E_ (K11 with K11gp17 anchor fused with KP32gp37 depolymerase; Fig. 4) is plated on K. pneumoniae 271 (host of KP32) (C). Download FIG S4, PDF file, 0.06 MB. Copyright © 2021 Latka et al. 2021 Latka et al. https://creativecommons.org/licenses/by/4.0/ This content is distributed under the terms of the Creative Commons Attribution 4.0 International license.

**FIG 5:**
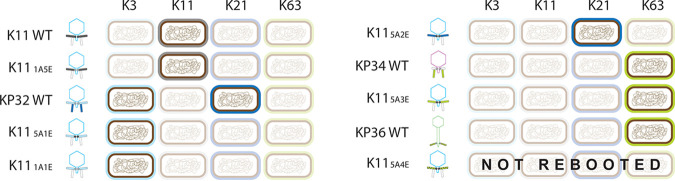
Specificity of the constructed synthetic phages against *Klebsiella* strains with different capsular serotypes. The construction of synthetic phages is presented in Table S4 with the respective codes. WT, wild-type phage.

10.1128/mBio.00455-21.8 TABLE S4 Overview of all synthetic phages constructed with the phage genome engineering platform in this study. The scaffold and the composition of the (chimeric) receptor binding proteins (RBPs) is given (X). The corresponding capsular serotype specificity of the synthetic phages is shown in Fig. 5. WT, wild-type phage. Download Table S4, PDF file, 0.06 MB. Copyright © 2021 Latka et al. 2021 Latka et al. https://creativecommons.org/licenses/by/4.0/ This content is distributed under the terms of the Creative Commons Attribution 4.0 International license.

Given the high identity (88%) between the anchor domains of the first RBPs of phage KP32 (1A) and K11 (5A), we first exchanged the K11gp17 anchor (5A) with the KP32gp37 anchor (1A) while conserving the native enzymatic domain of the first RBP of phage K11. Thus, a chimeric RBP (1A + 5E) was transplanted in a phage K11 scaffold, resulting in K11_1A5E_ (Fig. 4). Infective phage particles of K11_1A5E_ were successfully rebooted and were specific to K. pneumoniae 390 (capsular serotype K11) only, a wild-type K11 host (Fig. 5). These observations support that the anchor domain of the first RBP of phage KP32 is a true module with autonomous folding, independent of its neighboring domain, and has an autonomous function, i.e., attachment of the RBP to the phage tail. True modules can be most easily exchanged by horizontal transfer.

Subsequently, we aimed to modify the specificity of phage K11 by mimicking a horizontal transfer between phages K11 and KP32, both belonging to the *Przondovirus* genus. The enzyme domains of the first RBPs of phage K11 (5E) and phage KP32 (1E) were switched in a phage K11 scaffold, resulting in engineered phage K11_5A1E_ (Fig. 4). The rebooted modified phage was able to form plaques on K. pneumoniae 271 (serotype K3) and no longer on K. pneumoniae 390 (serotype K11) (Fig. 5 and Fig. S4). Analogously, the construction of phage K11 equipped with the anchor domain (1A) and the enzymatic domain (1E) of the first RBP of phage KP32 (K11_1A1E_; Fig. 4 and 5) led to the same shift to the K3 host, confirming again the exchangeability of the phage K11 and phage KP32 anchors.

Next, we transferred the second RBP of phage KP32 (KP32gp38; 2E) as a substitute for the enzymatic part of K11gp17 (5E) while conserving the wild-type anchor (5A). This modified phage (K11_5A2E_) became accordingly specific for the K21 capsular serotype while losing specificity for capsular serotype K11. Similarly, K11_5A3E_, possessing a chimera of the wild-type phage K11 anchor (5A) and the full depolymerase of phage KP34 (KP34gp57, 3E), was also successfully rebooted (Fig. S4), resulting in a host range switch from K. pneumoniae 390 (K11 serotype) to K. pneumoniae 77 and K. pneumoniae 486 (both K63 serotype). Phage K11 (*Przondovirus*) and phage KP34 (*Drulisvirus*) are both podoviruses at the morphological level (16, 35–37) but belong to different taxonomic groups. The enzyme domain of phage KP36 depolymerase (KP36gp50, 4E) shows a high amino acid similarity (43%) to phage KP34 depolymerase (3E) and shares specificity for capsular serotype K63. However, phage KP36 belongs to the *Drexlerviridae* family (siphoviruses). Any attempt to create the synthetic phage K11_5A4E_, bearing a chimeric fusion of the KP11gp17 anchor (5A) and the KP36 depolymerase (KP36gp50, 4E), was unsuccessful despite the fact that an enzymatically active chimera (5A + 4E) could be produced. Gibson assembly also was evaluated as an alternative approach to produce the synthetic phage genome but did not result in infective phages either, suggesting that a horizontal transfer between two distinct phage morphotypes (podo- and siphoviruses) is not trivial in this case.

## DISCUSSION

In this study, we aimed (i) to evaluate the triangular relationship between capsular serotype, host spectrum, and depolymerase specificity of *Klebsiella* phages and (ii) to mimic horizontal transfer on a laboratory scale as the major evolutionary driver of RBPs to switch the host spectrum of *Klebsiella* phages. A two-tiered approach was followed. First, we evaluated a set of chimeric RBPs for their functionality, which allowed us to identify transferable, functional units. Secondly, synthetic phages were produced with chimeric RBPs and their host spectrum was analyzed. The VersaTile technique was used as a click-and-play method to efficiently construct the coding sequences of chimeric proteins (32). The phage engineering platform based on gap repair cloning in S. cerevisiae and phage rebooting in electroporated E. coli 10G cells (33) was applied to create synthetic phages.

The construction of chimeric RBPs was based on the former confirmation of the enzymatic activity and specificity of the selected model RBPs with a depolymerase domain (8, 9, 31). All chimeric RBPs were enzymatically active with a specificity switch corresponding to the predicted enzymatic domain. The same specificity switches take place for synthetic phages bearing these chimeric RBPs, also confirming the role of the anchor domains. The long α-helix, inspired by the phage P22 RBP tertiary structure (21, 38), appeared to be a good predictor for delineation of the domains without the availability of crystal structures. These findings give experimental support for the evolution model of phage RBPs being created by horizontal transfers of enzymatic domains that can be plugged into different RBP architectures regardless of phage genus (*Przondovirus* versus *Drulisvirus*). Whereas the chimera combining the K11gp17 anchor with the enzyme domain of KP36gp50 (5A + 4E) is enzymatically active, using two different assembly techniques, we did not succeed in obtaining infective K11 phage particles with this enzymatic domain. This hints at an incompatible direct transfer between a siphovirus and a podovirus. However, a natural chimera combining a myovirus-like anchor and a podovirus-like enzymatic domain has been reported before (39). In addition, RBP shuffling has been successful across phage families for phage tail-like bacteriocins (40). Proper virion assembly may be hindered in synthetic phage K115A4E by differences in virion assembly between podovirus K11 and siphovirus KP36. In addition, many RBPs need a chaperone domain or additional tail fiber assembly protein for folding and trimerization. A putative tail assembly protein (KP36gp48) is identified two genes upstream of the KP36gp50 coding sequence; however, the chimeric RBP could be properly expressed as a functional recombinant protein, indicating correct folding. The putative tail assembly protein is predicted to be responsible for KP36gp49, which may act as a second side RBP, as in the case of some T5 viruses (DT57C and DT571) or the central tail tip (16); however, we cannot exclude a role in the proper assembly of KP36gp50 as well.

*Klebsiella* phage K11 is 91% identical to phage KP32 (with 88% query coverage) at the genome level and was eventually chosen as a scaffold for phage engineering. Notably, phage K11 belongs to *Przondovirus* (same subgroup as phage KP32; 16, 35–37), which, in turn, belongs to the *Autographiviridae* (former T7 supergroup). Ando et al. (33) reported before on the exchange of the first RBP of phage K11 (K11gp17) with the single tail fiber of E. coli phage T7 (gp17). Their initial attempts based on a simple exchange did not yield infective phages. The T7 phage tail is composed of a gp11 dodecamer, forming the adaptor, and a gp12 hexamer, forming the nozzle. The adaptor ring is responsible for the attachment of the preformed tail to the portal (gp8) of the prohead. The interaction between the tail and the six tail fibers (or RBPs) occurs at the interface of the adaptor and the nozzle (41, 42). The homology of the adaptor and nozzle between phages T7 and K11 is much lower than the homology of the portal protein between both phages. Therefore, the coding sequences of the adaptor (K11gp11), the nozzle (K11gp12), and the first RBP (K11gp17) were simultaneously exchanged in phage K11 for the equivalents of phage T7, resulting in K11 phage particles infecting E. coli. In contrast to the RBP of phage T7, the first RBP of phage KP32 could be simply transferred into phage K11 (88% similarity at the anchor level). Both phage K11 with a chimera of the phage K11 anchor and the phage KP32 enzymatic domain (K11_5A1E_), as well as phage K11 with the full RBP of phage KP32 (K11_1A1E_), result in infective phage particles without the need to swap the adaptor (gp11/gp31) and nozzle (gp12/gp32) proteins between K11 and KP32. This can be explained by the high identity of these proteins (97% [gp11/gp31] and 95% [gp12/gp32] at the amino acid level) between K11 and KP32. Thus, the anchors of both viruses can be used interchangeably.

Phage T7 encodes a single well-characterized RBP. At the time of the construction of K11-T7 chimeras (33) and the phage K11 chimeras in this work, phage K11 also was assumed to encode a single RBP. However, recent bioinformatic analyses revealed the presence of a second putative RBP in phage K11 (NCBI accession no. YP_002003831.1). This second RBP is predicted to dock on a T4gp10-like domain that comes immediately after the anchor domain of the first RBP (16). Phage K11 was screened against a panel of 79 *Klebsiella* serotypes (see Table S5 in the supplemental material), but no susceptible strain was found. The second RBP might have a capsular specificity not present in our collection and may target lipopolysaccharide or even a proteinaceous receptor. Alternatively, the enzymatic domain may have become inactivated due to mutations. Therefore, it remains unknown whether the second RBP of phage K11 is incorporated in the chimeric K11 phage particles. In this work, the predicted T4gp10-like domains are part of the enzyme module of KP32gp37 (1E) and K11gp17 (5E), which may offer docking sites for the second RBP of phage K11. However, KP32gp38 (2E), KP32gp57 (3E), and KP36gp50 (4E) have no predicted T4gp10-like domain. The modified phages K11_5A2E_ and K11_5A3E_ suggest that the incorporation of the second RBP is not essential to obtain infective phage particles. The same hypothesis is valid for phage K11 with gp11, gp12, and gp17 from phage T7, as T7gp17 is also lacking a T4gp10-like domain.

10.1128/mBio.00455-21.9 TABLE S5 List of strains and corresponding capsular serotypes used to search the second host of phage K11. Download Table S5, PDF file, 0.06 MB. Copyright © 2021 Latka et al. 2021 Latka et al. https://creativecommons.org/licenses/by/4.0/ This content is distributed under the terms of the Creative Commons Attribution 4.0 International license.

The modular build-up of phage RBP structures with recyclable building blocks responsible for the structural organization and receptor specificity, driven by intense horizontal transfers, provides an evolutionary highway for the rapid adaptation to the high diversity of K. pneumoniae capsules not necessarily constrained by taxonomic borders or different RBP architectures. With the recent maturation of synthetic biology tools for phage design (43–49), an increasing number of engineered phages can be constructed to gain further meaningful insights in the host recognition system by *Klebsiella* phage RBPs and the role of their domains in specificity and structural organization.

## MATERIALS AND METHODS

### Bacteriophages, bacterial hosts, yeast, and culture conditions.

Klebsiella pneumoniae bacteriophages KP32 (GenBank accession no. NC_013647.1), KP34 (NC_013649.2), and KP36 (NC_029099.1) were used from the collection of the Department of Pathogen Biology and Immunology, University of Wroclaw, Poland, and K11 (NC_011043.1) from the collection of the Synthetic Biology Group, Massachusetts Institute of Technology (Table 1). Podoviruses KP32 (and K11) and KP34 belong to separate genera of the *Autographiviridae* family (*Przondovirus* and *Drulisvirus*, respectively), whereas siphovirus KP36 belongs to the *Webervirus* genus of the *Drexlerviridae* family. All phages were plated using the soft agar (0.5% agar in tryptic soy broth [TSB]; bioMérieux, Marcy l’Etoile, France) method (50), poured on tryptic soy agar (TSA; bioMérieux, Marcy l’Etoile, France), and incubated for 18 h at 37°C. All *Klebsiella* strains listed in Table 2 were cultured in TSB (bioMérieux) or on TSA (bioMérieux) at 37°C.

**TABLE 1:** Phages used in this study

Phage	Accession no.	Taxonomy	Source^*a*^	Specificity to capsular serotype	K. pneumoniae host
KP32	NC_013647.1	*Autographiviridae*, *Studiervirinae*, *Przondovirus*	DPBI UWr	K3, K21	271, 358, 968
KP34	NC_013649.2	*Autographiviridae*, *Slopekvirinae*, *Drulisvirus*	DPBI UWr	K63	77, 486
KP36	NC_029099.1	*Drexlerviridae*, *Webervirus*	DPBI UWr	K63	77, 486
K11	NC_011043.1	*Autographiviridae*, *Studiervirinae*, *Przondovirus*	SBG MIT	K11	390

a DPBI UWr, Department of Pathogen Biology and Immunology, University of Wroclaw, Poland. SBG MIT, Synthetic Biology Group, Massachusetts Institute of Technology.

**TABLE 2:** K. pneumoniae strains used in this study

*Klebsiella* strain	Source^*a*^	Capsular serotype	Phage susceptibility
K. pneumoniae 271	DPBI UWr	K3	KP32
K. pneumoniae 390	SBG MIT	K11	K11
K. pneumoniae 358	DPBI UWr	K21	KP32
K. pneumoniae 968	DPBI UWr	K21	KP32
K. pneumoniae 77	DPBI UWr	K63	KP34, KP36
K. pneumoniae 486	DPBI UWr	K63	KP34, KP36

a DPBI UWr, Department of Pathogen Biology and Immunology, University of Wroclaw, Poland. SBG MIT, Synthetic Biology Group, Massachusetts Institute of Technology.

Escherichia coli strains used for plasmid propagation (TOP10; Invitrogen, Thermo Fisher Scientific, Waltham, MA) and protein expression [BL21(DE3), Invitrogen] were grown in standard lysogeny broth (LB; Biomaxima, Lublin, Poland) or on LB agar (LB supplemented with 1.5% bacteriological agar; VWR, Radnor, PA, USA). E. coli TOP10 transformed with a pVTEIII entry vector was grown on LB supplemented with 100 μg/ml ampicillin (Fisher Scientific, Thermo Fisher Scientific, Waltham, MA) and 5% sucrose (Fisher Scientific). E. coli TOP10 and BL21(DE3) transformed with a pVTD2 destination vector were grown on LB supplemented with 50 μg/ml kanamycin (Acros Organics, Thermo Fisher Scientific, Waltham, MA) and 5% sucrose (Fisher Scientific). For LB cultures of E. coli TOP10 and BL21(DE3) transformed with pEXP-5-CT/TOPO (Invitrogen), 100 μg/ml ampicillin was added. For phage genome assembly by gap repair cloning, Saccharomyces cerevisiae BY4741 (Thermo Scientific, Thermo Fisher Scientific, Waltham, MA) was used. Yeast cells were cultured in standard YPD medium (1% Bacto yeast extract [Becton, Dickinson, East Rutherford, NJ, USA], 2% Bacto peptone [Becton, Dickinson], 2% dextrose [VWR]) or in synthetic complemented minimal medium (SC complete, containing YNB plus nitrogen, complete supplement mixture [CSM]; Sunrise Science Products, Knoxville, TN) supplemented with 2% dextrose (VWR). For the selection of YAC-positive cells, SC-Leu (YNB plus nitrogen, CSM-Leu; Sunrise Science Products) supplemented with 2% dextrose (VWR) was used. A 2% agar supplementation was applied for solid medium (yeast culture-grade agar; Sunrise Science Products). Yeast cells were grown at 30°C for 24 to 72 h. For electroporation with phage genomes captured in a YAC, E. coli 10G (Lucigen, LGC Ltd., Teddington, Great Britain) cells were used.

### Plasmids.

Tiles were cloned in the pVTEIII vector and subsequently assembled in the pVTD2 vector (32). For cloning and expression of the coding sequences for the enzyme domains and anchor and truncated RBPs, commercially available vectors pEXP-5-CT/TOPO and pEXP-5-NT/TOPO (Invitrogen) were used. Some proteins (KP32gp37, KP32gp38, KP34gp57 and KP36gp50) have been cloned and expressed before (8, 9, 31, 56). For synthetic assembly in yeast, the yeast centromere vector pRS415 with LEU2 marker (ATCC 87520) was used.

### Construction of chimeric RBPs.

The VersaTile technique was applied to make chimeric RBPs similar to how the technique was used to construct chimeric lysins (32). The method follows a two-step approach: (i) a repository of all building blocks (i.e., tiles) is created with VersaTile cloning, and (ii) selected tiles are combined in a single assembly reaction using VersaTile shuffling (Fig. 1). Tiles were prepared by PCR using Phusion DNA polymerase (Thermo Fisher Scientific, Waltham, MA) and specifically designed primers (see Table S1 in the supplemental material). The flanking position tags determine the position of the tile in the final assembly. Gel extraction of the amplicons was performed using the GeneJET gel extraction kit (Thermo Fisher Scientific). Subsequently, the purified amplicons were inserted into the entry vector pVTEIII by type IIs cloning (SapI/T4 DNA ligase). The tile encoding a hexahistidine purification tag was prepared by cassette hybridization. Briefly, a mixture of primers (His tag F and R; Table S1) was incubated for 2 min at 95°C and then gradually cooled down. *Pfu* polymerase (Thermo Fisher Scientific) was added (10 min at 72°C) to fill in the single overhanging strands. The cassette was purified using the GeneJET PCR purification kit (Thermo Fisher Scientific) and inserted into the pVTEIII entry vector as described above. Present BsaI recognition sites in the coding sequences of tiles were mutated before using specific primers (Table S1). In these cases, the tile of interest was assembled from two (KP34gp57; 3E) or three (KP36gp50; 4E) amplicons in one restriction (SapI)/ligation (T4 DNA ligase) reaction, simultaneously inserting the assembled sequence in the pVTEIII entry vector. After enzyme inactivation of the assembly reaction (50°C for 5 min and 65°C for 20 min), restriction/ligation mixtures were used for the transformation of chemocompetent E. coli TOP10 cells by heat shock. Positive constructs, verified by colony PCR, were Sanger sequenced by LGC Genomics (Berlin, Germany) using appropriate primers (Table S1). Plasmid DNA of tiles ready for a VersaTile shuffling reaction was isolated from overnight cultures using the GeneJET plasmid miniprep kit (Thermo Fisher Scientific). For the assembly reaction, a tile for positions 1, 2, and 3 (each 50 ng) and pVTD2 (100 ng) vector were added to a mixture of BsaI (10 U), T4 DNA ligase (1 U), 10× ligation buffer, and ultrapure water (total volume of 20 μl). Fifty cycles of restriction/ligation were conducted (2 min at 37°C and 3 min at 16°C) followed by enzyme inactivation for 5 min at 50°C and 5 min at 80°C. Restriction/ligation mixtures were used for the transformation of chemocompetent E. coli TOP10 cells by heat shock. Correct plasmids verified by Sanger sequencing were used for E. coli BL21(DE3) transformation for chimeric protein expression.

10.1128/mBio.00455-21.5 TABLE S1 Primers used in this study (sequence 5′→3′). The codes of the specific domains refer to Fig. 2. Download Table S1, PDF file, 0.2 MB. Copyright © 2021 Latka et al. 2021 Latka et al. https://creativecommons.org/licenses/by/4.0/ This content is distributed under the terms of the Creative Commons Attribution 4.0 International license.

### Cloning of coding sequences of reference depolymerase domains and anchor and truncated RBPs.

Coding sequence fragments were amplified by PCR (Table S1) with *Pfu* DNA polymerase (Thermo Fisher Scientific), followed by postamplification 3′ A-overhang addition by DreamTaq polymerase (Thermo Fisher Scientific). Subsequently, amplicons were cloned into the pEXP-5-CT/TOPO vector (Invitrogen) with a C-terminal histidine tag according to the manufacturer's conditions. Transformation of chemocompetent E. coli TOP10 cells for plasmid propagation and isolation was followed by Sanger sequencing. Corrected constructs were used for E. coli BL21(DE3) transformation and subsequent protein expression.

### Protein expression and visualization.

The recombinant E. coli BL21(DE3) cells were grown in LB supplemented with appropriate antibiotic at 37°C with agitation until the optical density at 600 nm reached 0.5. The culture was induced by the addition of 0.1 mM isopropyl-β-d-thiogalactopyranoside (IPTG; Bio-Connect BV), followed by 18 h of incubation at 20°C. Cultures were pelleted by centrifugation (5,000 × *g*, 10 min, 4°C), resuspended in lysis buffer containing 20 mM NaH_2_PO_4_-Na_2_HPO_4_ (Acros Organics), 0.5 M NaCl (Acros Organics), pH 7.4, and lysed by three cycles of freeze-thawing followed by sonication. For spot tests, cell lysate was used. For analysis of protein fractions, SDS-PAGE (180 V for approximately 1 h) was performed with 12% polyacrylamide gels (51). Coomassie brilliant blue R-250 (Bio-Rad, Hercules, CA, USA) staining was applied for protein visualization.

### Activity spot test and specificity analysis.

Bacterial lawns were prepared by pouring 1 ml of overnight *Klebsiella* culture on TSA plates. Ten microliters of cell lysate was spotted on a dried bacterial lawn with 10 μl of lysis buffer and BL21(DE3) lysate (with an empty plasmid) as controls. Plates were incubated at 37°C overnight.

### Phage engineering.

Phage engineering was performed by gap-repair cloning in S. cerevisiae, with some modifications (33). KAPA HiFi DNA polymerase (Kapa Biosystems, Roche, Basel, Switzerland) was used to amplify phage genome fragments with overlapping ends, including the intended genome modification (Table S1). The first and last phage genome fragments were equipped with sequences homologous to the termini of linearized YAC. PCR products were used as such for further steps of genome assembly. To produce the linearized YAC vector, the shuttle YAC vector pRS415 (propagated in E. coli TOP10) was digested by PvuII (New England BioLabs, Ipswich, MA) and XbaI (New England BioLabs). The fragment (2,910 nt long) enabling propagation in yeast cells (CEN/ARS) and selection (LEU2, LEU2 promoter) was amplified with KAPA HiFi DNA polymerase using specific primers (Table S1) and digested by DpnI (New England BioLabs), and the amplicon was purified by gel extraction (Zymoclean gel DNA recovery; Zymo Research, Freiburg im Breisgau, Germany).

Competent S. cerevisiae BY4741 cells were prepared as described previously (33). PCR amplicons covering the phage genome, including the fragment corresponding to the genome modification, were mixed in equal concentrations. The PCR amplicon mixture (Table S2) and 20 ng of linear YAC fragment was added to competent cells. The mixture was vortexed, followed by a 45-min-long incubation at 42°C and centrifugation (13,000 × *g*; 30 s). Transformed cells were resuspended in 200 μl SC complete, incubated for 1 h at 30°C, plated on SC-Leu, and grown at 30°C for 72 h.

10.1128/mBio.00455-21.6 TABLE S2 Summary of primer pairs used to create the amplicons to construct the genome of the modified K11 phages. The coded name (boldface) of each modified phage comprises the scaffold, while the subscript indicates the chimeric RBP that replaces the first RBP (K11gp17). The first two letters represent the anchor and the following two letters indicate the enzymatic part (according to nomenclature given in Fig. 2A). For each modified genome, a set of six overlapping amplicons had to be produced with the corresponding primers listed in Table S1. Figure 4 gives a schematic overview of each modified phage. Download Table S2, PDF file, 0.01 MB. Copyright © 2021 Latka et al. 2021 Latka et al. https://creativecommons.org/licenses/by/4.0/ This content is distributed under the terms of the Creative Commons Attribution 4.0 International license.

Precultures of successful transformants were started from single colonies in 5 ml of SC-Leu and incubated at 30°C for 72 h. DNA extraction was performed according to the manufacturer’s instructions using the YeaStar genomic DNA kit (Zymo Research). Extracted DNA was used for electroporation of 25 μl E. coli 10G cells (2-mm-gap electroporation cuvette [Bio-Rad]; standard settings were 2.5 kV, 25 μF, 200 Ω; Gene Pulser Xcell [Bio-Rad]). Cells were immediately recovered into 1 ml of SOC medium (Lucigen) and incubated at 37°C for 3 h with agitation. To kill the cells and to release the rebooted phages, 100 μl chloroform (VWR) was added, followed by vortexing and spinning down. Supernatant (0.5 ml) was mixed with 200 μl of the appropriate logarithmically growing host strain (Table 2) in 3 ml top agar (TSB and 0.5% agar, 55 to 60°C) and plated on TSA. Plates were checked for plaques after incubation at 37°C for 18 h.

Attempts were undertaken to produce recombinant phage K11_5A4E_ using Gibson assembly (Gibson assembly master mix; NEB) for genome construction covered by 6 fragments (Tables S1 and S2) as described before (52, 53), followed by E. coli 10G electroporation, but no infective phage particles were observed.

### Data availability.

All supporting data, sequences, and accession numbers are available in the supplemental material.
