# Supplementary material for: Engineering the Modular Receptor-Binding Proteins of Klebsiella Phages Switches Their Capsule Serotype Specificity
Source: mBio. 2021 May 4;12(3):e00455-21. doi: 10.1128/mBio.00455-21 (PMC8262889; doi:10.1128/mBio.00455-21)
Supplement: FIG S1 [file mbio.00455-21-sf001.pdf]

**Supplementary material**

**A**

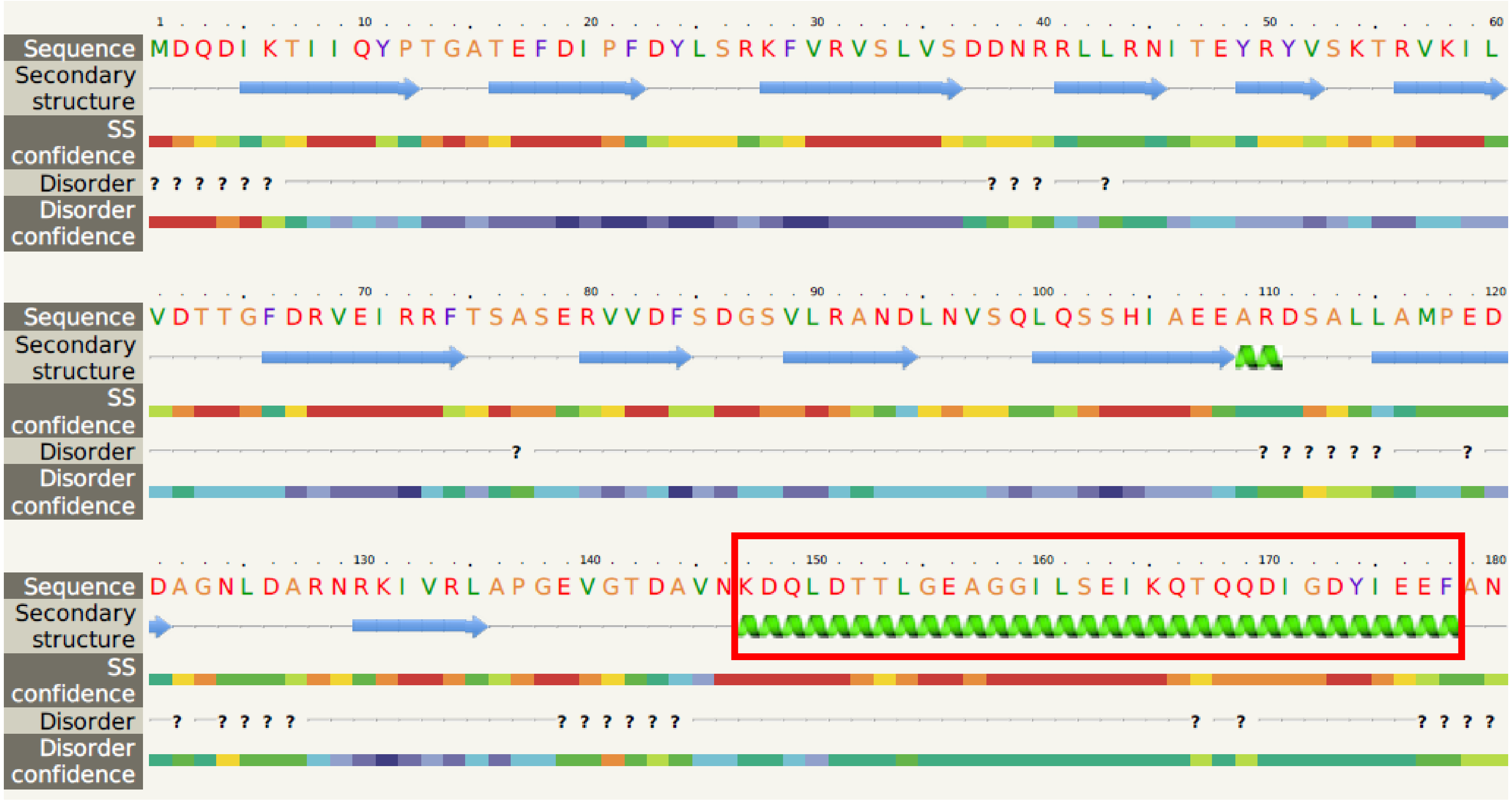

B

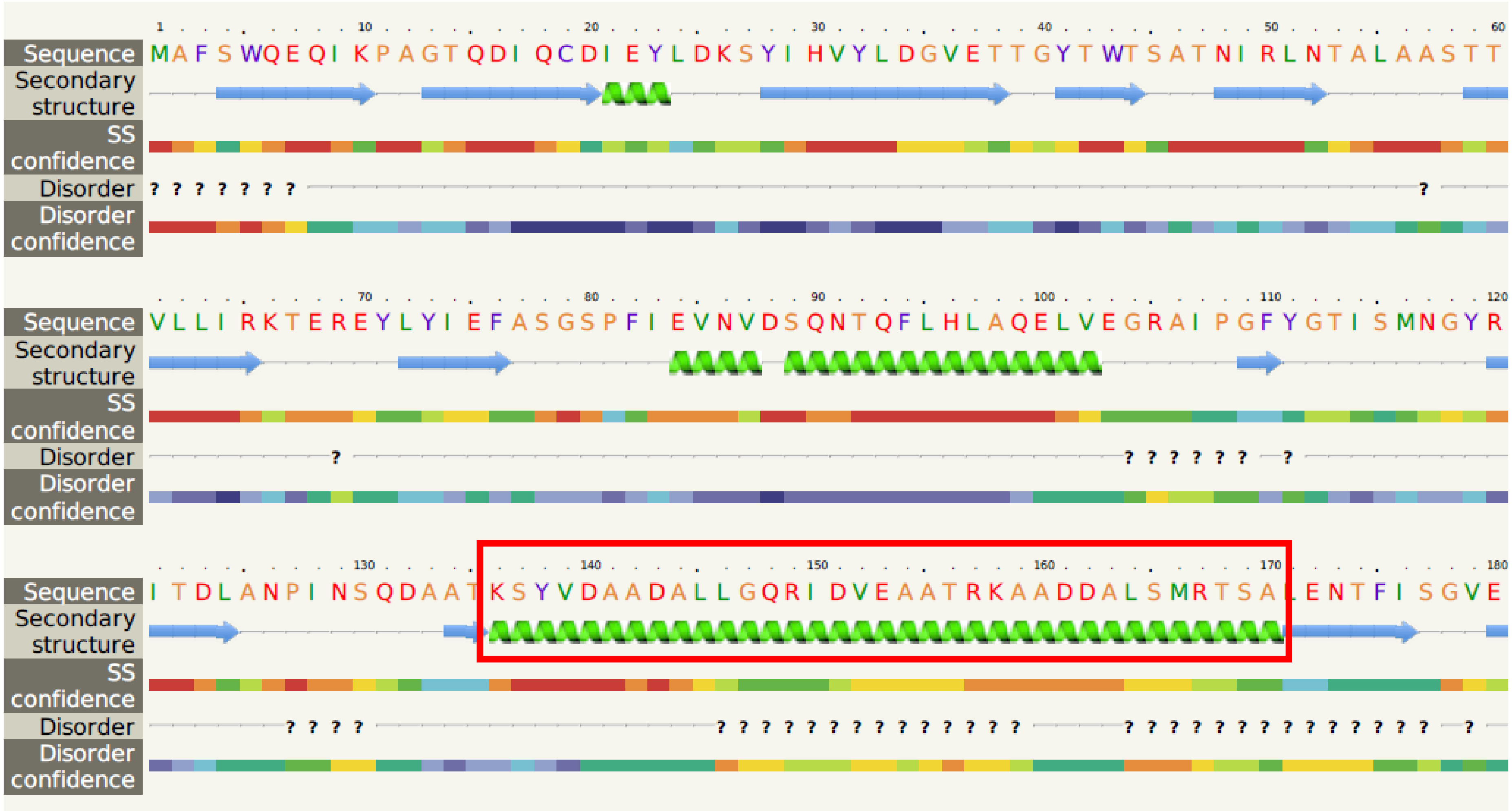

Sequence
Secondary structure
SS confidence
Disorder
Disorder confidence
1 10 20 30 40 50 60
MAFSWQEQIKPAGTQDIQCDIEYLDKSYIHVYLDGVETTGYTWTSATNIRLNTALAASTT
70 80 90 100 110 120
VLLIRKTEREYLYIEFASGSPFIEVNVDSQNTQFLHLAQELVEGRAIPGFYGTISMNGYR
130 140 150 160 170 180
ITDLANPINSQDAATKSYVDAADALLGQRIDVEAATRKAADDALSMRTSALENTFISGVE

C

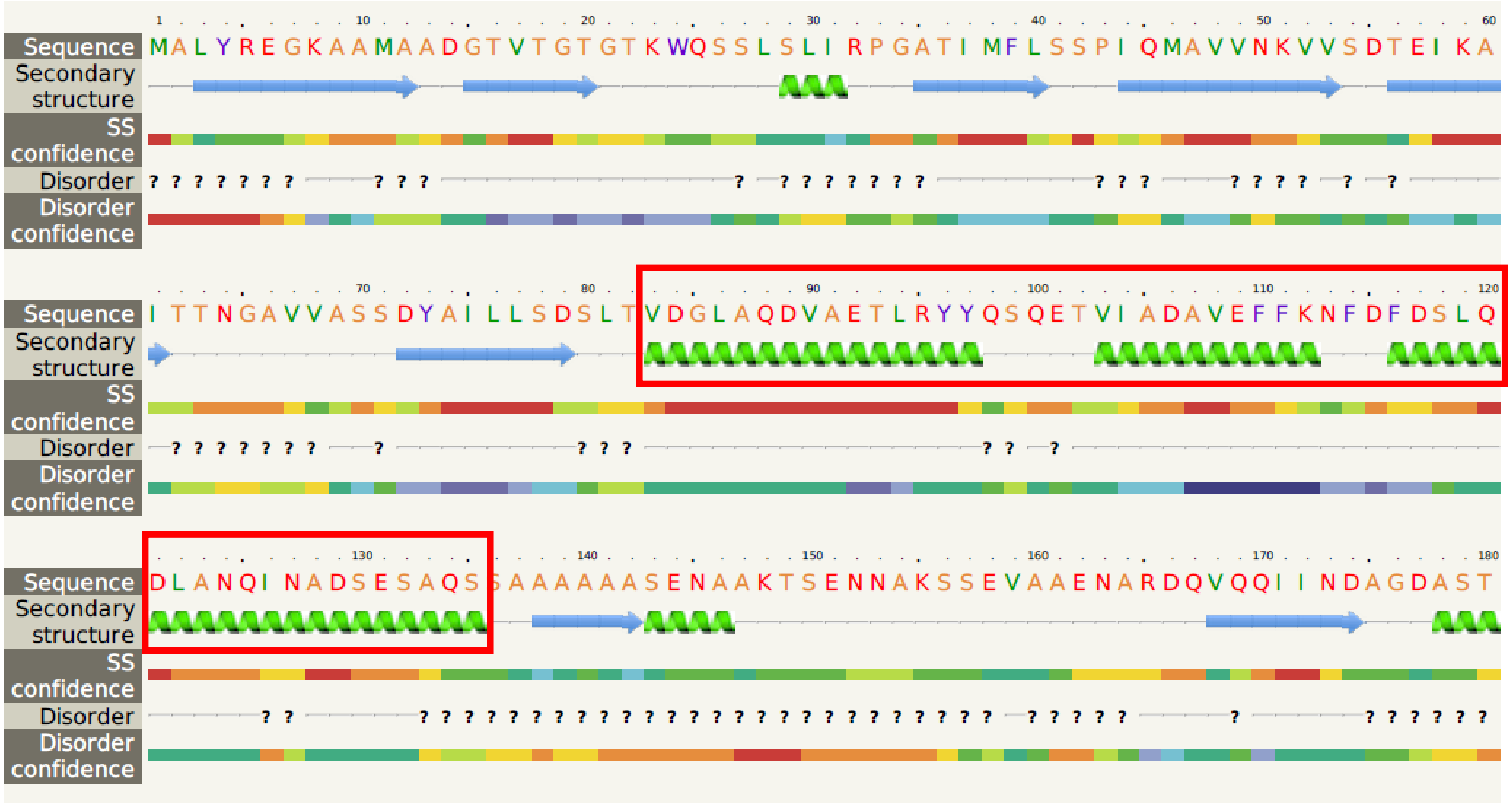

Sequence
Secondary structure
SS confidence
Disorder
Disorder confidence
MALYREGKAAMAADGTVTGTGTKWQSSLSLIRPGATIMFLSSPIQMAVVNKVVSDTEIKA
ITTNGAVVASSDYAILLSDSLTVDGLAQDVAETLRYYQSQETVIADAVEFFKNFDFDSLQ
DLANQINADSESAQSSAAAAAASENAAKTSENNAKSSEVAAENARDQVQQIINDAGDAST

**D**

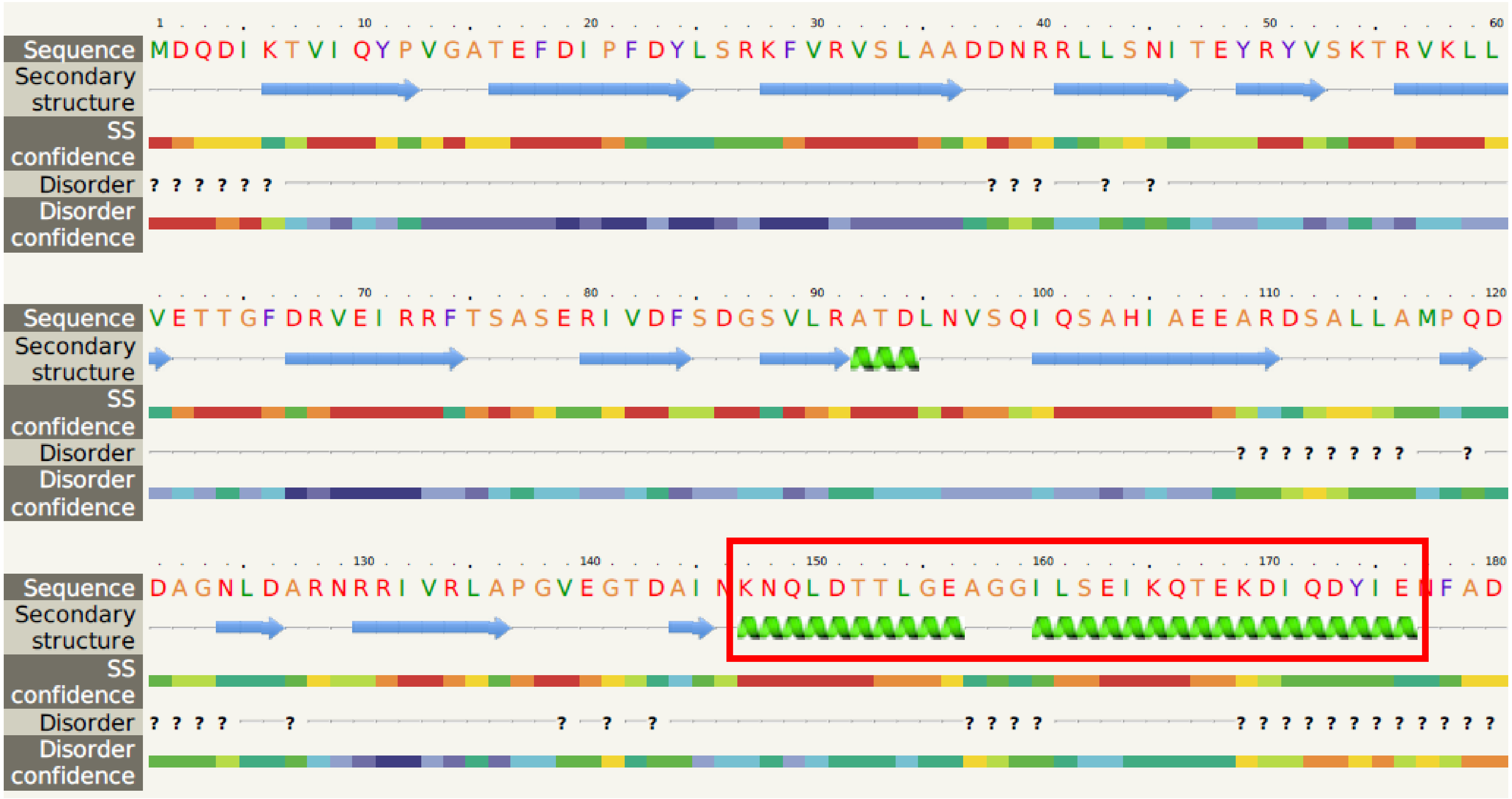


**Figure S1.** Identification of long α-helices that separate anchor domains. The consensus secondary structures of the N-termini of KP32gp37 (A), KP34gp49 (B), KP36gp50 (C) and K11gp17 (D) were predicted using Phyre2 (Kelley et al. 2015). Long α-helixes that are hypothesized to physically separate the N-terminal dome-like domain and the enzymatic domain, are indicated in red frames.
