# Supplementary material for: Engineering the Modular Receptor-Binding Proteins of Klebsiella Phages Switches Their Capsule Serotype Specificity
Source: mBio. 2021 May 4;12(3):e00455-21. doi: 10.1128/mBio.00455-21 (PMC8262889; doi:10.1128/mBio.00455-21)
Supplement: FIG S2 [file mbio.00455-21-sf002.pdf]

Supplementary material

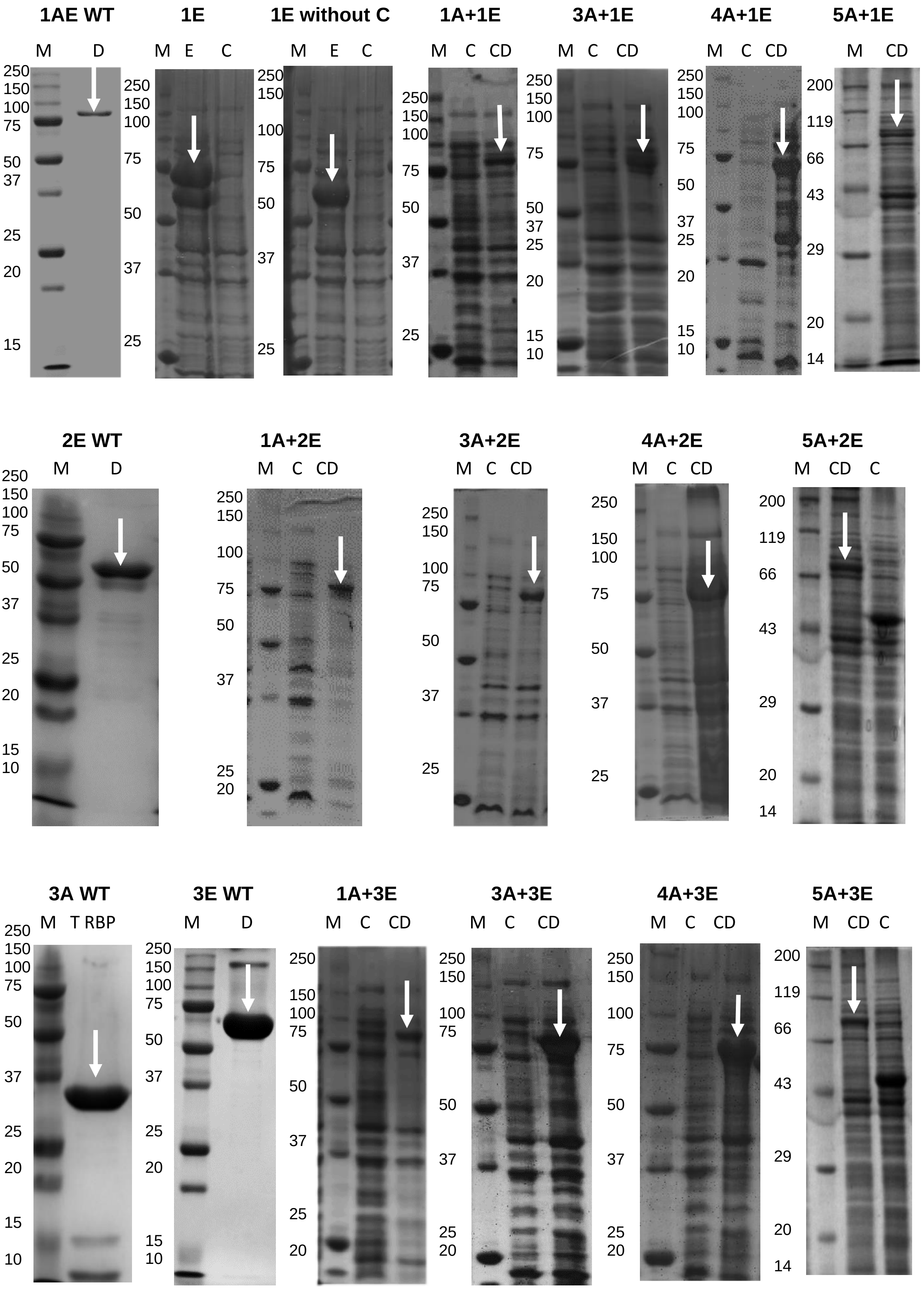

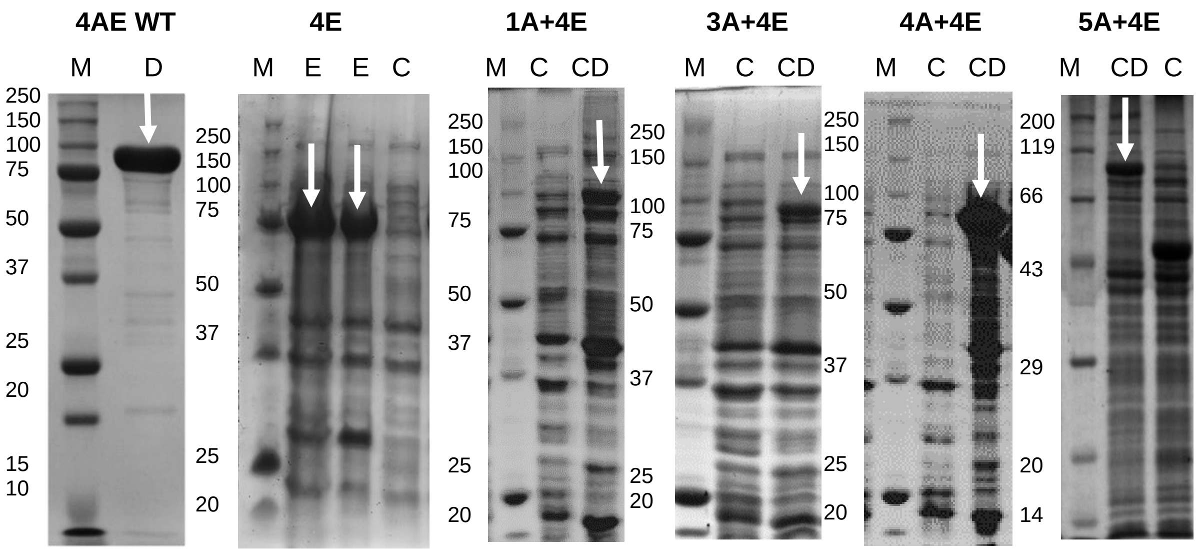

Figure S2. Overexpression of chimeric depolymerases (CD), truncated receptor binding protein (T RBP), enzyme domain of depolymerases (E), and wild-type depolymerases (D) compared to BL21(DE3) cells transformed with plasmid without insert as a control (C). Wild-type proteins were purified and purified protein is shown, while for all other proteins the lysate is shown as indicated in Table S3. M indicates the molecular mass marker (Precision Plus Protein Unstained Standards, BioRad or Roti Mark Standard, Carl Roth) is indicated. Definition of the code and the respective molecular weights can be found in the Table S3.
