# Supplementary material for: Engineering the Modular Receptor-Binding Proteins of Klebsiella Phages Switches Their Capsule Serotype Specificity
Source: mBio. 2021 May 4;12(3):e00455-21. doi: 10.1128/mBio.00455-21 (PMC8262889; doi:10.1128/mBio.00455-21)
Supplement: FIG S3 [file mbio.00455-21-sf003.pdf]

**Supplementary material**

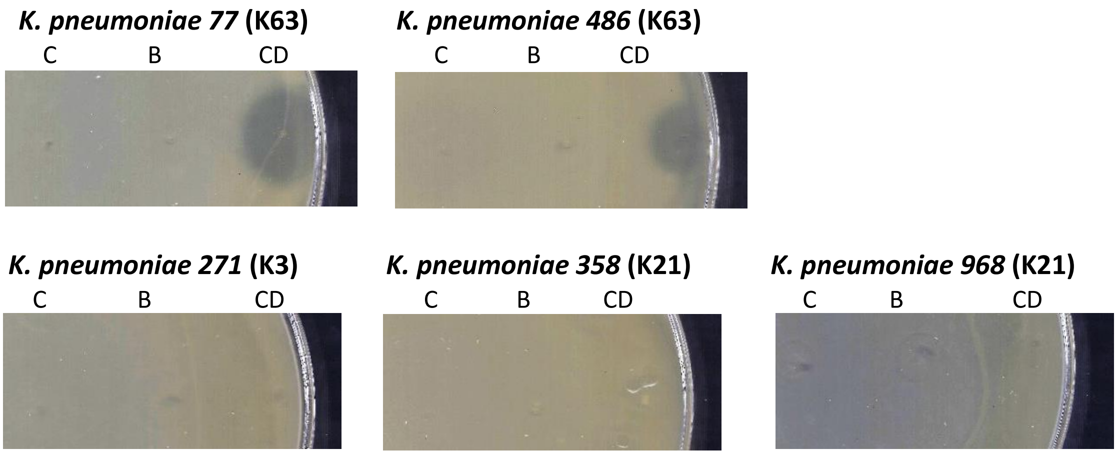


**Figure S3.** Typing of a chimeric depolymerase on *K. pneumoniae* strains with different capsular serotypes, illustrated here for the chimeric depolymerase 1A+3E (KP32gp37 anchor fused with the KP34gp57 depolymerase). Volumes of 10 µl lysate containing either the chimeric depolymerase or one of the controls were spotted on bacterial lawns of five different strains with three different serotypes (CD – chimeric depolymerase 1A+3E, C – bacterial lysate with empty vector, B – lysis buffer used for lysate formation). The chimeric depolymerase remains specific to capsular serotype K63 similar to the specificity of the enzymatic domain from KP34gp57 (3E).
