# Supplementary material for: Engineering the Modular Receptor-Binding Proteins of Klebsiella Phages Switches Their Capsule Serotype Specificity
Source: mBio. 2021 May 4;12(3):e00455-21. doi: 10.1128/mBio.00455-21 (PMC8262889; doi:10.1128/mBio.00455-21)
Supplement: TABLE S3 [file mbio.00455-21-st003.pdf]

Supplementary material

Table S3. Composition of all chimeras with expected molecular weights (MW, kDa). In the case of KP32gp37 (1AE), which has a C-terminal autocleaving chaperone domain, the expected MW of the cleaved protein is given as well as the MW of the autocleaved moiety (12.3 kDa). Soluble protein fractions (SPF) were examined to check overexpression of chimeric depolymerases/wild-type (WT) depolymerase/enzymatic domains, expect when protein overexpression was not visible in the SPF. In these cases, the total protein fraction (TPF), which also comprises insoluble proteins, was examined instead (Figure S2). Fractions (SPF/TPF) marked with “V” are visualised with SDS-PAGE (Figure S2). “X” indicates the presence of a particular domain in the chimeric depolymerase/wild-type (WT) depolymerase/enzymatic domain. “+” between domains in the code name means that the chimeric protein is the result of VersaTile assembly. When there is no “+” between the anchor (A) and enzyme (E) domain, the wild-type (WT) depolymerase was prepared as in the native sequence without scar between the anchor and enzyme domain, whereas VersaTile assembled chimeras have an intervening linker of two amino acids between both domains, resulting from the position tag.

Code	N-terminus				C-terminus				SDS-PAGE		
	KP32	KP34	KP36	K11	KP32		KP34	KP36			
	gp37 anchor (1A)	gp49 anchor (3A)	gp50 anchor (4A)	gp17 anchor (5A)	gp37 enzyme (1E)	gp38 whole (2E)	gp57 whole (3E)	gp50 enzyme (4E)	TPF	SPF	MW (kDa)
**1AE WT**	X				X					V	97.85 – 12.3
**1E**					X				V		75.32 – 12.3
**1E without C**					X (truncated)				V		64.04
**1A + 1E**	X				X					V	96.57 – 12.3
**3A + 1E**		X			X					V	95.31 – 12.3
**4A + 1E**			X		X					V	90.92 – 12.3
**5A + 1E**				X	X					V	96.43 – 12.3
**2E WT**						X				V	62.79
**1A + 2E**	X					X			V		82.93
**3A + 2E**		X				X				V	81.67
**4A + 2E**			X			X				V	77.28
**5A + 2E**				X		X				V	82.66
**3A WT**		X (full-length)								V	33.82
**3E WT**							X			V	67.74
**1A + 3E**	X						X			V	87.88
**3A + 3E**		X					X			V	86.62
**4A + 3E**			X				X			V	82.23
**5A + 3E**				X			X			V	87.61
**4AE WT**			X					X		V	94.98
**4E**								X		V	79.51
**1A + 4E**	X							X		V	100.75
**3A + 4E**		X						X		V	99.49
**4A + 4E**			X					X		V	95.10
**5A + 4E**				X				X		V	100.61

“truncated” – the protein possesses the enzyme domain with a deleted chaperone domain as indicated in Table S1 (aa 179-769).
“full length” – the protein does not only possess the anchor domain but covers the full length sequence of KP34gp49.
