# Supplementary material for: Engineering the Modular Receptor-Binding Proteins of Klebsiella Phages Switches Their Capsule Serotype Specificity
Source: mBio. 2021 May 4;12(3):e00455-21. doi: 10.1128/mBio.00455-21 (PMC8262889; doi:10.1128/mBio.00455-21)
Supplement: FIG S4 [file mbio.00455-21-sf004.pdf]

Supplementary material

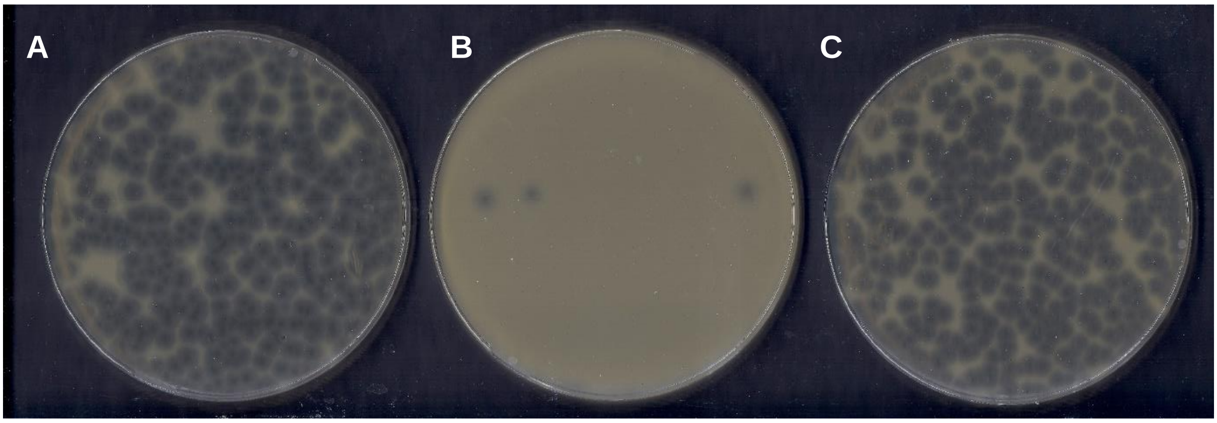

Figure S4. Representative plaques of rebooted phages using the phage engineering platform. Assembled wild-type K11 is plated on *K. pneumoniae* 390 (host of K11) (A), $K11_{5A3E}$ (K11 with K11gp17 anchor fused with KP34gp57 depolymerase, Figure 4) is plated on *K. pneumoniae* 77 (host of KP34) (B), $K11_{5A1E}$ (K11 with K11gp17 anchor fused with KP32gp37 depolymerase, Figure 4) is plated on *K. pneumoniae* 271 (host of KP32) (C).
