# Supplementary material for: Engineering the Modular Receptor-Binding Proteins of Klebsiella Phages Switches Their Capsule Serotype Specificity
Source: mBio. 2021 May 4;12(3):e00455-21. doi: 10.1128/mBio.00455-21 (PMC8262889; doi:10.1128/mBio.00455-21)
Supplement: TABLE S4 [file mbio.00455-21-st004.pdf]

Supplementary material

Table S4. Overview of all synthetic phages constructed with the phage genome engineering platform in this study. The scaffold and the composition of the (chimeric) receptor binding proteins (RBPs) is given (“X”). The corresponding capsular serotype specificity of the synthetic phages is shown in Figure 5. WT – wild-type phage.

Code	Scaffold				RBP						
					N-terminus		C-terminus				
	K11	KP32	KP34	KP36	K11gp17 anchor	KP32gp37 anchor	K11gp17 enzyme	KP32gp37 enzyme	KP32gp38 whole	KP34p57 whole	KP36gp50 enzyme
**K11 WT**	x				x		x				
**$K11_{1A5E}$**	x					x	x				
**KP32 WT**		x				x		x	x		
**$K11_{5A1E}$**	x				x			x			
**$K11_{1AE}$**	x					x		x			
**$K11_{5A2E}$**	x				x				x		
**KP34 WT**			x							x	
**$K11_{5A3E}$**	x				x					x	
**KP36 WT**				x							x
**$K11_{5A4E}$**	x				x						x
