# Supplementary material for: Engineering the Modular Receptor-Binding Proteins of Klebsiella Phages Switches Their Capsule Serotype Specificity
Source: mBio. 2021 May 4;12(3):e00455-21. doi: 10.1128/mBio.00455-21 (PMC8262889; doi:10.1128/mBio.00455-21)
Supplement: TABLE S5 [file mbio.00455-21-st005.pdf]

**Supplementary material**

**Table S5.** List of strains and corresponding capsular serotypes used to search the second host of phage K11.

| K type | Species | Name | Susceptibility to phage K11 | |
|---|---|---|---|---|
| **K1** | *K. pneumoniae* | 52.144 | - | - |
| **K2** | *K. pneumoniae* | 52.145 | - | - |
| **K3** | *K. pneumoniae* | 52.146 | - | - |
| **K4** | *K. pneumoniae ozaenae* | 52.211 T | - | - |
| **K5** | *K. pneumoniae ozaenae* | 52.212 | - | - |
| **K6** | *K. pneumoniae ozaenae* | 52.213 | - | - |
| **K7** | *K. pneumoniae* | 52.205 | - | - |
| **K8** | *K. pneumoniae* | 52.206 | - | - |
| **K9** | *K. pneumoniae* | 52.207.1 | - | - |
| | | 52.207.2 | - | - |
| **K10** | *K. pneumoniae* | 52.214 | - | - |
| **K11** | *K. pneumoniae* | 52.215 | + | + |
| **K12** | *K. pneumoniae* | 52.216 | - | - |
| **K13** | *K. pneumoniae* | 52.217 | - | - |
| **K14** | *K. pneumoniae* | 52.218 | - | - |
| **K15** | *K. pneumoniae* | 52.21 | - | - |
| **K16** | *K. pneumoniae* | 52.220 | - | - |
| **K17** | *K. pneumoniae* | 52.221 | - | - |
| **K18** | *K. pneumoniae* | 52.222 | - | - |
| **K19** | *K. pneumoniae* | 52.223 | - | - |
| **K20** | *K. pneumoniae* | 52.224 | - | - |
| **K21** | *K. pneumoniae* | 52.225 | - | - |
| | | 52.968 | - | - |
| | | 52.358 | - | - |
| **K22** | *K. pneumoniae* | 52.226 | - | - |
| **K23** | *K. pneumoniae* | 52.228 | - | - |
| **K24** | *K. pneumoniae* | 52.229 | - | - |
| **K25** | *Raoultella planticola* | 52.230 | - | - |
| **K26** | *K. pneumoniae* | CIP 53.6 | - | - |

| | | | | |
|---|---|---|---|---|
| **K27** | *K. pneumoniae* | CIP 52.232 | - | - |
| **K28** | *K. pneumoniae* | CIP 52.233 | - | - |
| **K29** | *K. pneumoniae* | CIP 52.234 | - | - |
| **K30** | *K. pneumoniae* | CIP 52.235 | - | - |
| **K31** | *K. pneumoniae* | CIP 52.231 | - | - |
| **K32** | *K. pneumoniae* | CIP 53.7 | - | - |
| **K33** | *K. pneumoniae* | CIP 53.8 | - | - |
| **K34** | *K. pneumoniae* | CIP 53.9 | - | - |
| **K35** | *K. pneumoniae* | CIP 53.10 | - | - |
| **K36** | *K. pneumoniae* | CIP 53.11 | - | - |
| **K37** | *K. pneumoniae* | CIP 53.12 | - | - |
| **K38** | *K. pneumoniae* | CIP 53.13 | - | - |
| **K39** | *K. pneumoniae* | CIP 53.14.1 | - | - |
| | | CIP 53.14.2 | - | - |
| | | CIP 53.14.3 | - | - |
| **K40** | *K. pneumoniae* | CIP 53.15 | - | - |
| **K41** | *K. pneumoniae* | CIP 53.16 | - | - |
| **K42** | *K. pneumoniae* | CIP 53.17 | - | - |
| **K43** | *K. pneumoniae* | CIP 53.19 | - | - |
| **K44** | *K. pneumoniae* | CIP 53.20 | - | - |
| **K45** | *K. pneumoniae* | CIP 53.21 | - | - |
| **K46** | *K. pneumoniae* | CIP 53.22 | - | - |
| **K47** | *K. pneumoniae* | CIP 53.23 | - | - |
| **K48** | *K. pneumoniae* | CIP 53.24 | - | - |
| **K49** | *K. pneumoniae* | CIP 52.199 | - | - |
| **K50** | *K. pneumoniae* | CIP 52.200 | - | - |
| **K51** | *K. pneumoniae* | CIP 52.201 | - | - |
| **K52** | *K. pneumoniae* | CIP 53.25 | - | - |
| **K53** | *K. pneumoniae* | CIP 53.26 | - | - |
| **K54** | *K. pneumoniae* | CIP 53.27 | - | - |
| **K55** | *K. pneumoniae* | NCTC 9175 | - | - |
| **K56** | *K. pneumoniae* | NCTC 9176 | - | - |
| **K57** | *K. pneumoniae* | NCTC 9177 | - | - |
| **K58** | *K. pneumoniae* | NCTC 9178.1 | - | - |
| | | NCTC 9178.2 | - | - |

| | | | | |
|---|---|---|---|---|
| **K59** | *Raoultella planticola* | NCTC 9179 | - | - |
| **K60** | *K. pneumoniae* | NCTC 9180 | - | - |
| **K61** | *K. pneumoniae* | NCTC 9181.1 | - | - |
| | | NCTC 9181.2 | - | - |
| **K61** | *K. pneumoniae* | CIP 108293.1 | - | - |
| | | CIP 108283.2 | - | - |
| **K62** | *K. pneumoniae* | NCTC 9182 | - | - |
| **K63** | *K. pneumoniae* | NCTC 9183.1 | - | - |
| | | NCTC 9183.2 | - | - |
| **K64** | *K. pneumoniae* | NCTC 9184 | - | - |
| | | CIP 80.47 | - | - |
| **K65** | *Raoutella terrigena* | NCTC 9185 | - | - |
| **K66** | *K. oxytoca* | NCTC 9186 | - | - |
| **K67** | *Klebsiella* sp. | NCTC 9187 | - | - |
| **K68** | *Klebsiella* sp. | NCTC 9188 | - | - |
| **K69** | *Klebsiella* sp. | NCTC 9189 | - | - |
| **K70** | *K. oxytoca* | NCTC 10261 | - | - |
| **K71** | *K. pneumoniae* | NCTC 10262 | - | - |
| **K72** | *Raoutella planticola* | NCTC 10263 | - | - |
| **K74** | *K. oxytoca* | NCTC 11355 | - | - |
| **K79** | *K. oxytoca* | NCTC 11356 | - | - |
| **K80** | *K. pneumoniae* | NCTC 11357 | - | - |
| **K81** | *K. pneumoniae* | NCTC 11358 | - | - |
| **K82** | *K. pneumoniae* | NCTC 11359 | - | - |
