# Supplementary material for: Engineering the Modular Receptor-Binding Proteins of Klebsiella Phages Switches Their Capsule Serotype Specificity
Source: mBio. 2021 May 4;12(3):e00455-21. doi: 10.1128/mBio.00455-21 (PMC8262889; doi:10.1128/mBio.00455-21)
Supplement: TABLE S1 [file mbio.00455-21-st001.pdf]

Supplementary material

Table S1. Primers used in this study (sequence 5'→3'). The codes of the specific domains refer to Figure 2.

Name	Sequence	Purpose	Accession number and amplified region
**Primers to prepare tiles in position 1, 2 and 3**			
***1A F**	GTGGCTCTTCCAGAGGTCTC**ACCATG**GACCAAGATATTAAAACAATCATTCAGTAC	To clone KP32gp37 anchor (1A) as tile in position 1	YP_003347555.1 1-178 aa
***1A R**	ATAGCTCTTCCCTTGGTCTC**TGAACC**AAACTCCTCGATGTAATCCCCG		
***1E F**	GTGGCTCTTCAAGAGGTCTC**GGTTCT**GCCAACGGAACGACTTACCTTAAG	To clone KP32gp37 enzyme (1E) as tile in position 2	YP_003347555.1 179-869 aa
***1E R**	GTGGCTCTTCACTTGGTCTC**GTAAGA**TTTGTAGGTCAGGCCGAGCTTAC		
***2E F**	GTGGCTCTTCAAGAGGTCTC**GGTTCT**TTAGACAATTTCAATCAGCCG	To clone KP32gp38 (2E) as tile in position 2	YP_003347556.1 1-576 aa
***2E R**	GTGGCTCTTCACTTGGTCTC**GTAAGA**TGATACGAATGCCCTTACTCG		
***3A F**	GTGGCTCTTCCAGAGGTCTC**ACCATG**GCATTCAGCTGGCAAGAACAAATC	To clone KP34gp49 anchor (3A) as tile in position 1	YP_003347643.1 1-170 aa
***3A R**	ATAGCTCTTCCCTTGGTCTC**TGAACC**AGCGGACGTACGCATACTCAAAG		
***3E F**	GTGGCTCTTCAAGAGGTCTC**GGTTCA**GCACTCACTAAACTAGTAGATGCAGG	To clone KP34gp57 (3E) as tile in position 2	YP_003347651.1 1-630 aa
***3E R**	GTGGCTCTTCACTTGGTCTC**GTAAGA**ACCAGTGAGTTCAGATGGAGC		
***4A F**	GTGGCTCTTCCAGAGGTCTC**ACCATG**GCACTATACAGAGAAGGCAAAGC	To clone KP36gp50 anchor (4A) as tile in position 1	YP_009226011.1 1-135 aa
***4A R**	ATAGCTCTTCCCTTGGTCTC**TGAACC**TGATTGTGCAGATTCAGAGTCTGC		
***4E F**	ATAGCTCTTCAAGAGGTCTC**GGTTCA**AGCGCTGCGGCTGCTGCTGCGTCTGAAAATG	To clone KP36gp50 enzyme (4E) as tile in position 2	YP_009226011.1 136-883 aa
***4E R**	GTGGCTCTTCACTTGGTCTC**GTAAGA**TGCCGTCAAATCTTCTAACTGAAGATTCATTC		
***5A F**	GTGGCTCTTCCAGAGGTCTC**ACCATG**GACCAAGACATTAAAACAGTCATTC	To clone K11gp17 anchor (5A) as tile in position 1	YP_002003830.1 1-178 aa
***5A R**	ATAGCTCTTCCCTTGGTCTC**TGAACC**AAAGTTCTCGATGTAATCCTGAATG		
***His-F**	GTGGCTCTTCCAGAGGTCTC**TCTTAC**CATCATCACCATCACCATTGA	To clone His-tag as tile in position 3	6xHis 1-6 aa
***His-R**	GTGGCTCTTCCCTTGGTCTC**ATACTT**TCAATGGTGATGGTGATGATG		
**Primers to remove BsaI recognition sites from the 3E and 4E coding sequences (between brackets, the pairing primer is indicated)**			
**3E BsaI removal F**	TTTGCTCTTCTGCACAAAGCTACCCCAGCC	To mutate nt 549 in KP34p57 (3E) (with 3E R)	YP_003347651.1 557-1893 bp
**3E BsaI removal R**	TTTGCTCTTCGTGCACTACCAA**A**GTCTCCGTAGTGTC	To mutate nt 549 in KP34p57 (3E) (with 3E F)	YP_003347651.1 1-561 bp
**4E BsaI removal F1**	TTTGCTCTTCATGCAAGAGA**T**CAAGTTCAGCAGATC	To mutate nt 498 to in KP36gp50 (4E) (with 4E BsaI removal R2)	YP_009226011.1 488-2465 bp
**4E BsaI removal R1**	TTTGCTCTTCTGCATTCTCCGCAGCCAC	To mutate nt 498 in KP36gp50 (4E) (with 4E F)	YP_009226011.1 1-492 bp

**4E BsaI removal F2**	TTTGCTCTTCGCTATTATCACTTCCTTCCTTTCG	To mutate nt 2457 in KP36gp50 (4E) (with 4E R)	YP_009226011.1 2461-2652 bp
**4E BsaI removal R2**	TTTGCTCTTCATAGCCGA**A**GTCTCAAGCTCATTTG	To mutate nt 2457 in KP36gp50 (4E) (with 4E BsaI removal F1)	YP_009226011.1 488-2465 bp
**Primers for clone analysis and sequencing**			
**T7_promoter_F**	TAATACGACTCACTATAGGG	Clone analysis and sequencing of pVTD2 and pEXP5-CT/TOPO vector	NA
**T7_terminal_primer_R**	TAGTTATTGCTCAGCGGTGG	Clone analysis and sequencing of pVTD2 and pEXP5-CT/TOPO vector	NA
**pVTEIII-F**	TCTTTCCTGCGTTATCCC	Clone analysis and sequencing of pVTEIII	NA
**pVTEIII-R**	CATGAGCGGATACATATTTG	Clone analysis and sequencing of pVTEIII	NA
**1E mid sequencing**	GCATCCTGTGCCGTAACACC	KP32gp37 745-764 sequencing	YP_003347555.1 NA
**3E mid sequencing**	GGTCGGTAGTGTACGGC	KP34p57 742-758 sequencing	YP_003347651.1 NA
**4E mid sequencing**	GCCCCTCACATTCAAGCC	KP36gp50 861-878 sequencing	YP_009226011.1 NA
**Primers for TOPO cloning of the coding sequences of depolymerase domains and truncated receptor binding proteins (RBPs)**			
**1E WT F**	GCCAACGGAACGACTTACCTTAAGAACATC	To clone KP32gp37 (1E)	YP_003347555.1 179-869 aa
**1E WT R**	TTTGTAGGTCAGGCCGAGCTTACG		
**1E without C WT F**	GCCAACGGAACGACTTACCTTAAGAACATC	To clone KP32gp37 enzyme (1E) without C-terminus	YP_003347555.1 179-769 aa
**1E without C WT R**	GGTAACGTTAGGGGAGTTCGCCAGATAG		
****1AE WT F**	ATGGACCAAGATATTAAAACAATCATTCAGTAC	To clone KP32gp37 (1AE)	YP_003347555.1 1-869 aa
****1AE WT R**	TTATTTGTAGGTCAGGCCGAGCTTACG		
****2E WT F**	ATGTTAGACAATTTCAATCAGCCG	To clone KP32gp38 (2E)	YP_003347556.1 1-576 aa
****2E WT R**	TGATACGAATGCCCTTACTCGG		
**3A WT F**	ATGGCATTCAGCTGGCAAGAAC	To clone KP34p49 (3A)	YP_003347643.1 1-307 aa
**3A WT R**	TGAAGCCAGTACCCCGCTG		
****3E WT F**	ATGGCACTCACTAAACTAGTAGATGCAGG	To clone KP34p57 (3E)	YP_003347651.1

****3E WT R**	ACCAGTGAGTTCAGATGGAGCAAAGCAGC		1-630 aa
**4E WT F**	AGCGCTGCGGCTGCTGCTGCGTCTGAAAATG	To clone KP36gp50 (4E)	YP_009226011.1 136-883 aa
**4E WT R**	TGCCGTCAAATCTTCTAACTGAAGATTCATTC		
****4AE WT F**	ATGGCACTATACAGAGAAGGCAAAG	To clone KP36gp50 (4AE)	YP_009226011.1 1-883 aa
****4AE WT R**	TGCCGTCAAATCTTCTAACTGAAG		
**Primers for assembly of the wild-type phage K11 genome and modifications therein**			
**YAC-1**	CCTGTACTTCCTTGTTCATGTGTGTTCAAA	Amplification of a linearized YAC-fragment encoding CEN/ARS, LEU2, LEU2 promoter on pRS415 template	ATCC 87520 5391-2309 bp
**YAC-2**	ATAAACAAATAGGGGTTCCGCGCACATTTC		
**K11-20**	TTTGAACACACATGAACAAGGAAGTACAGGTCTCACAGTTTACACTTTTGGTTATCCCCC	Amplification of genome fragments to assemble the wildtype K11 genome	NC_011043.1 1-10000 bp
**K11-21**	ATTAGAAGTCATCGTCTTCTTCGGCTTCGC		
**K11-22**	AGCGGACGAATCTCGCAGCCGTAAACCTCA	Amplification of genome fragments to assemble the wildtype K11 genome	NC_011043.1 9900-19990 bp
**K11-23**	TCATCACCTTCGAGGGCCTTAAGGGCTGAC		
**K11-24**	ATTGCCGCATGGTCAGCCCTTAAGGCCCTC	Amplification of genome fragments to assemble the wildtype K11 genome	NC_011043.1 19950-29979 bp
**K11-25**	CATCGTGTCCTTGAACACATCGTACCCATC		
**K11-26**	CGGGGACGCTGCTGAGGCTCAGATTCAGAA	Amplification of genome fragments to assemble the wildtype K11 genome	NC_011043.1 29880-41181 bp
**K11-27**	GAAATGTGCGCGGAACCCCTATTTGTTTATAGGGACACAGAGACATCAACATATAGTGTC		
**K11-29**	GACCTCCTTAAGTTGAATAGGAGGGAAACCGGAAACCAATCGGTCTCCCTATAGTGTGTC	Amplification of the fragment to replace 5A with 1A in the K11 genome (with K11-26)	NC_011043.1 29880-33757 bp
**K11-30**	CGTGGTGTCATCTGCAAACTCCTCGATGTAATCCCCGATATCCTG	Amplification of the fragment to replace 5A with 1A in the K11 genome (with KP32-15)	YP_003347555.1 1-178 aa
**K11-31**	TACATCGAGGAGTTTGCAGATGACACCACGTCTCTCAAGGGAATC	Amplification of the fragment to replace 5A with 1A in the K11 genome (with K11-27)	NC_011043.1 33755-41181 bp
**K11-32**	GGAGAACCTCATGTTAGACAATTTCAATCAGGAGACAACATGCTGAATGATTTAAACCAA	Amplification of the fragment to replace 5E with 1E in the K11 genome (with K11-27)	NC_011043.1 35852-41181 bp
**K11-34**	GCTCATCATGAAAATCTATGAAGCCAACG	PCR/ sequencing (with K11-38); Colony PCR (with 1AE WT R/2E WT R/ 3E WT R/ 4AE WT R)	NC_011043.1 NA
**K11-38**	GGTAAGGTGTCCAACCGCTTCTTCAACG	PCR/ sequencing (with K11-34)	NC_011043.1

			NA
**K11-45**	GGAGACAACATGCTGAATGATTTAAAC	Amplification of the fragment to replace 5E with 2E/3E/4AE in the K11 genome (with K11-27)	NC_011043.1 35852-41181 bp
**K11 1E F**	AAGGACATTCAGGATTACATCGAGAACTTTGCCAACGGAACGACTTACCTTAAGAAC	Amplification of the fragment to replace 5E with 1E in the K11 genome (with KP32 17)	YP_003347555.1 179-869 aa
**K11 5A R**	AAAGTTCTCGATGTAATCCTGAATGTC	Amplification of the fragment to replace 5E with 2E/3E/4AE in the K11 genome (with K11-26)	NC_011043.1 29880-33757 bp
**K11 3E F**	AAGGACATTCAGGATTACATCGAGAACTTTATGGCACTCACTAAACTAGTAGATG	Amplification of the fragment to replace 5E with 3E in the K11 genome	YP_003347651.1 1-630 aa
**K11 3E R**	TTGGTTTAAATCATTCAGCATGTTGTCTCCCTAACCAGTGAGTTCAGATGGAG		
**K11 4E F**	AAGGACATTCAGGATTACATCGAGAACTTTAGCGCTGCGGCTGCTGCTG	Amplification of the fragment to replace 5E with 4E in the K11 genome	YP_009226011.1 136-883 aa
**K11 4E R**	TTGGTTTAAATCATTCAGCATGTTGTCTCCTTATGCCGTCAAATCTTCTAACTGAAG		
**K11 2E F**	AACAGACCGAGAAGGACATTCAGGATTACATCGAGAACTTTATGTTAGACAATTTCAATC	Amplification of the fragment to replace 5E with 2E in the K11 genome	YP_003347556.1 1-576 aa
**K11 2E R**	GCCTCGTGGTTGGTTTAAATCATTCAGCATGTTGTCTCCTTATGATACGAATGCCCTTAC		
**K11 1AE F**	GGTTTCCCTCCTATTCAACTTAAGGAGGTC	Amplification of the fragment to replace 5AE with 1AE in the K11 genome	YP_003347555.1 1-869 aa
**K11 1AE R**	TGATTGAAATTGTCTAACATGAGGTTCTCC		
**Gibson 1 F**	TCTCACAGTTTACACTTTTGGTTATCCCCC	Amplification of genome fragments to assemble the wild-type K11 genome by Gibson assembly	NC_011043.1 1-10020 bp
**Gibson 1 R**	CTTTGGCCCCATAGCCCGCCATTAGAAGTCATCGTCTTCTTCG		
**Gibson 2 F**	AGAAGACGATGACTTCTAATGGCGGGCTATGGGG	Amplification of genome fragments to assemble the wild-type K11 genome by Gibson assembly	NC_011043.1 9981-20020 bp
**Gibson 2 R**	CGTAACTTGAGGTTAGCCAAGTTGAGGTCGTCATCACC		
**Gibson 3 F**	AAGGTGATGACGACCTCAACTTGGCTAACCTCAAGTTACG	Amplification of genome fragments to assemble the wild-type K11 genome by Gibson assembly	NC_011043.1 19981-30020 bp
**Gibson 3 R**	TCTTCCTTGGAGGGAACATAAGAGTTCCAGCGAGTCG		
**Gibson 4 F**	CCCCGACTCGCTGGAACTCTTATGTTCCCTCCAAGGAAGAC	Amplification of genome fragments to assemble the wild-type K11 genome by Gibson assembly	NC_011043.1 29980-41181 bp
**Gibson 4 R**	AGGGACACAGAGACATCAACATATAGTGTC		
**Colony YAC F**	CACCTGGCAAAACGACGATCTTC	Colony PCR (with C K11YAC R)	NA

**Colony YAC R**	GCACGTGATGAAAAGGACCCAG	Colony PCR (with C K11YAC F)	NA
**Colony K11YAC F**	CACTAAGAGCCAACATAAGGAGGAC	Colony PCR (with C YAC R)	NA
**Colony K11YAC R**	CTAATCCATATTGTTAAAGAGCGGTGCTTC	Colony PCR (with C YAC F)	NA
**1AE WT R**	TTATTTGTAGGTCAGGCCGAGCTTACG	Colony PCR (with K11-34)	NA
**2E WT R**	TGATACGAATGCCCTTACTCGG	Colony PCR (with K11-34)	NA
**3E WT R**	ACCAGTGAGTTCAGATGGAGCAAAGCAGC	Colony PCR (with K11-34)	NA
**4E WT R**	TGCCGTCAAATCTTCTAACTGAAG	Colony PCR (with K11-34)	NA

Underlined sequences correspond to the SapI and BsaI recognition sites, the position tag (6 nt) or the modified nucleotide (1 nt) are indicated in bold.

NA – not applicable.

*These primers extend the coding sequence from outwards to inwards with 1) the recognition site for SapI (NEB); 2) a restriction site for cloning into entry vector (pVTEIII); 3) the recognition site for BsaI (Life Technologies); 4) a position-specific tag for the VersaTile assembly reaction.

**Primers used previously to produce wild-type depolymerases (Majkowska-Skrobek et al., 2016, Majkowska-Skrobek et al., 2018, Squeglia et al., 2020 and Latka & Drulis-Kawa 2020), which were used in this study as control of wild-type depolymerase specificity.
