# Supplementary material for: Engineering the Modular Receptor-Binding Proteins of Klebsiella Phages Switches Their Capsule Serotype Specificity
Source: mBio. 2021 May 4;12(3):e00455-21. doi: 10.1128/mBio.00455-21 (PMC8262889; doi:10.1128/mBio.00455-21)
Supplement: TABLE S2 [file mbio.00455-21-st002.pdf]

Supplementary material

Table S2. Summary of primer pairs used to create the amplicons to construct the genome of the modified K11 phages. The coded name (bold) of each modified phage comprises the scaffold, while the subscript indicates the chimeric RBP that replaces the first RBP (K11gp17). The first two letters represent the anchor and the following two letters indicate the enzymatic part (according to nomenclature given in Figure 2A). For each modified genome, a set of six overlapping amplicons had to be produced with the corresponding primers listed in Table S1. Figure 4 gives a schematic overview of each modified phage.

K11$_{1A5E}$
K11-20+K11-21
K11-22+K11-23
K11-24+K11-25
K11-26+K11-29
KP32-15+K11-30
K11-31+K11-27

K11$_{1A1E}$
K11-20+K11-21
K11-22+K11-23
K11-24+K11-25
K11-26+K11-29
KP32-15+KP32-17
K11-32+K11-27

K11$_{5A1E}$
K11-20+K11-21
K11-22+K11-23
K11-24+K11-25
K11-26+K11 5A R
K11 1E F+KP32-17
K11-32+K11-27

Gibson K11$_{5A4E}$
Gibson 1 F+ Gibson 1 R
Gibson 2 F+ Gibson 2 R
Gibson 3 F+ Gibson 3 R
Gibson 4 F+ K11 5A R
K11 4E F+K11 4E R
K11-45 + Gibson 4 R

K11$_{5A3E}$
K11-20+K11-21
K11-22+K11-23
K11-24+K11-25
K11-26+K11 5A R
K11 3E F+K11 3E R
K11-45+K11-27

K11$_{5A2E}$
K11-20+K11-21
K11-22+K11-23
K11-24+K11-25
K11-26+K11 5A R
K11 2E F+K11 2E R
K11-45+K11-27

K11$_{5A4E}$
K11-20+K11-21
K11-22+K11-23
K11-24+K11-25
K11-26+K11 5A R
K11 4E F+K11 4E R
K11-45+K11-27
